# Dielectric and Magnetic Spherical Hollow Shells Subjected to a dc or Low-Frequency ac Field of *Any* Spatial Form: Complete Theoretical Survey of All Scalar and Vector Physical Entities, Including the Depolarization Effect

**DOI:** 10.3390/ma19081638

**Published:** 2026-04-19

**Authors:** Petros Moraitis, Kosmas Tsakmakidis, Norbert M. Nemes, Dimosthenis Stamopoulos

**Affiliations:** 1 Department of Physics, School of Science, National and Kapodistrian University of Athens, Zografou Panepistimioupolis, 15784 Athens, Greece; morapet@phys.uoa.gr (P.M.); ktsakmakidis@phys.uoa.gr (K.T.); 2 Instituto de Ciencia de Materiales de Madrid, CSIC, 28049 Madrid, Spain; nmnemes@fis.ucm.es; 3 Departamento de Física de Materiales, Universidad Complutense de Madrid, E-28040 Madrid, Spain

**Keywords:** spherical hollow shells, depolarization effect, depolarization factor, demagnetizing effect, demagnetizing factor, shielding applications, cloaking applications, dielectrophoresis, magnetophoresis

## Abstract

Dielectric and magnetic spherical hollow shells are employed in many applications as standard building units. These structures are commonly subjected to size reduction to obtain a high surface area/volume ratio, a property that is in favor of specific applications. However, the size reduction enhances the importance of physical mechanisms that originate from surfaces, such as the depolarization effect. Here we tackle the problem of dielectric and magnetic spherical hollow shells, consisting of a linear, homogeneous and isotropic parent material, subjected to an external potential, $U_{ext}\left(r\right)$, of any spatial form (either dc (static) or ac of low-frequency (quasistatic limit)). By applying the method-of-linear-recursive-solution (MLRS) to the Laplace equation, we calculate analytically the internal, $U_{int}\left(r\right)$, and total, $U_{tot}\left(r\right)$, potentials in respect to the external one, $U_{ext}\left(r\right)$. From $U_{int}\left(r\right)$ and $U_{tot}\left(r\right)$ we calculate all relevant scalar and vector physical entities of interest. The MLRS unveils straightforwardly the existence of two distinct depolarization factors, $N_{l}=l/\left(2l+1\right)$ and $N_{l+1}=\left(l+1\right)/\left(2l+1\right)$, both depending on the degree, l, however not on the order, m, of the mode of the external potential, $U_{ext}^{\left(l,m\right)}\left(r\right)$. These depolarization factors, $N_{l}$ and $N_{l+1}$, originate from the outer, r=b, and inner, r=a, surfaces and are accompanied by two extrinsic susceptibilities, $\chi_{e,l}^{ext}=\chi_{e}/\left(1+N_{l}\chi_{e}\right)$ and $\chi_{e,l+1}^{ext}=\chi_{e}/\left(1+N_{l+1}\chi_{e}\right)$, respectively. Importantly, $N_{l}+N_{l+1}=1$, irrespective of the degree, l, as it should. The properties of spherical hollow shells are investigated through analytical modeling and detailed simulations, with emphasis on application-relevant scenarios including resonance phenomena in scattering, quantitative materials characterization, and shielding/distortion. The generic MLRS strategy provides a flexible and reliable route for analyzing depolarization processes in other dielectric and magnetic building-unit geometries encountered in practice.

## 1. Introduction

Dielectric and magnetic materials have been studied extensively, both theoretically and experimentally, in modern physics. Typical structures of these materials in the form of regular shapes, e.g., plates, spheres and cylinders, serve as cornerstone building units for a vast number of applications. Depending on the nature of application, these building units can have a complicated or simple inner structure. Most commonly, they come in the form of a solid (whether homogeneous, multilayered or gradient) or of a hollow shell.

Spherical dielectric and magnetic building units are employed in scattering and shielding applications, e.g., invisibility cloaks [1,2,3,4,5], to model the response and enable the manipulation of biological cells, colloids and polymers during sorting e.g., dielectrophoresis [6,7,8,9]; in therapeutic biomedicine, e.g., blood purification and drug delivery [10,11,12,13]; and in electromagnetic wave prevention and control, e.g., microwave absorption [14,15,16,17,18]; to name just a few. Referring to scattering and shielding applications, in [1] a three-dimensional magnetic cloak was evidenced to operate under static and quasi-static conditions (i.e., from static magnetic fields up to the noticeable frequency of 250 kHz). This was demonstrated by means of a layered spherical structure consisting of an inner superconducting hollow shell in contact to an outer magnetic one. The dynamic performance of the hybrid spherical structure was verified by experimental measurements of the nearby field, which were in good agreement with relevant simulations ([1] presents a useful brief review of the relevant literature). In [2] the authors considered the response of a sphere of radially anisotropic permittivity dyadic for both cases of an applied electric field, static and dynamic. Among other interesting results, the authors showed that for appropriate values of the permittivity, the sphere could perform efficiently as an invisibility cloak, leaving the electric field of the outside space unperturbed. This was proven by means of rather simple quasistatic calculations and by results obtained with standard Mie scattering theory. In [3] the authors investigated both computationally and experimentally a three-dimensional layered spherical structure consisting of an inner metallic hollow shell in contact to an outer magnetic one. This hybrid spherical structure could operate at room temperature by harnessing the conventional diamagnetic behavior of normal metals (instead of the advanced behavior of the superconductors used in [1]). This spherical building unit exhibited nice performance as a quasistatic magnetic cloak (maximum field disturbance ratio 0.5%) for a wide frequency range starting from 5 kHz up to 250 kHz ([3] presents a useful brief review of the relevant literature). In [4] the conditions of perfect cloaking of a three-dimensional object were investigated, over the visible spectrum, for any angle of incidence and for any polarization of light. In this effect, it was proposed that any fundamental restriction can be lifted by means of fast-light-media-based cloaks. Both analytic calculations and simulations were recruited for the demonstration of highly efficient three-dimensional cloaking over the visible spectrum. In [5] a bi-layered concentric spherical structure was studied consisting of two different parent materials having both dielectric and conducting properties. The structure was embedded in a distinct medium having both dielectric and conducting properties, as well. The authors obtained analytical conditions for successful cloaking for both the transient and steady states when an electric field was applied. These analytical results were supported by means of simulations with FEM that confirmed the cloaking effectiveness. Regarding dielectrophoresis, in [6] colloidal dielectric particles were investigated upon application of ac and dc electric fields. The precise manipulation of particles motion was surveyed for a range of parameters such as the intensity and frequency of the electric field, and the geometry of the electrodes. The results on the manipulation of both nanoparticles and microparticles by means of planar electrodes were reported. The principles of operation of droplet-based microfluidic chips, biosensors, and devices were reviewed for particles in the form of diluted suspensions, as well. In [7] the technique of dielectrophoresis was reported for the case where the electrodes responsible for the production of the desired non-uniform electric field are not in contact with the sample fluid. In this approach the electrodes are capacitively coupled to a fluidic channel so that the externally applied electric field to these electrodes ultimately induces the desired electric field in the fluidic channel. The versatility of this technique was demonstrated experimentally through fabrication of a microfluidic device and the assessment of its performance upon cell manipulation. In [8] the technique of dielectrophoresis was thoroughly reviewed for the manipulation (e.g., trapping, focusing, sorting, and separation) of colloidal, inert and biological particles (e.g., healthy and cancer cells, bacteria, proteins, DNA, etc.) suspended in a buffer medium. Various parameters were reviewed (e.g., particles’ charge, specific geometry of the employed microfluidic device, dielectric properties of both the buffer medium and the particles, to name just a few). The experimental practice was reviewed in close comparison to theory for both cases of ac and dc dielectrophoresis. The contactless dielectrophoresis, a versatile and contamination-safe variation in the standard technique, was reviewed as well. In [9] the authors investigated the isolation of tumor cells from a blood sample, aiming to assist the timely diagnosis and treatment of cancer. To this end, they designed and fabricated a microfluidic device that subsequently was employed in experiments on human breast cancer cells (MCF-7 cells). Then, the tumor cells were conjugated to iron oxide magnetic nanoparticles (magnetite, Fe_3_O_4_) via a specific antibody (EpCAM) for the enhancement of their isolation by a Neodymium permanent magnet producing a field of 0.51 T. These experiments documented that the employed hybrid microfluidic device that combined an inertial and a magnetic method in a single chip performed excellently, exhibiting a recovery rate and purity of recovered tumor cells up to 95% and 93%, respectively. In respect to therapeutic biomedical applications, in [10] the authors investigated the utilization of magnetic nanoparticles in blood purification. Specifically, they reported on the comparison of standard dialysis experiments with magnetically assisted dialysis ones. Both were performed on the removal of a typical toxin, namely Homocysteine (Hcy), from a saline solution. For the magnetically assisted dialysis experiments, conjugates of iron oxide magnetic nanoparticles (magnetite/maghemite, Fe_3_O_4_/Fe_2_O_3_) with a serum albumin (BSA) were employed. The experimental results evidenced that the magnetically assisted dialysis performed much better than the standard method. Moving to drug delivery applications, in [11] highly sophisticated spherical units were synthesized, namely double- and triple-shelled hollow microporous spheres of a sulfonated organic network. Their performance on drug delivery was assessed on a comparative basis to that of single-shelled hollow spheres. It was proven that the additional compartmentalization improved the overall drug loading and release efficiency. Also, in [12] uniform monodisperse double-shelled silica hollow spherical shells with controllable inner structure have been successfully synthesized via an alternating acid/base selective etching strategy. A model anti-cancer drug, namely mitoxantrone, was chosen as a test molecule in drug delivery and release experiments. It was evidenced that the double-shelled spherical units exhibit higher drug-loading and drug-release capacities in comparison to conventional single-shelled silica hollow spherical shells. The in vitro cellular internalization of the double-shelled spherical units was documented through labeling with fluorescein isothiocyanate. The in vitro study with liver carcinoma cells proved that the mitoxantrone-loaded double-shelled spherical units have both reduced toxicity and enhanced therapeutic efficiency. In [13] chitosan-coated poly-methacrylic-acid hollow spherical shells were prepared through an effective self-assembly process. Doxorubicin loading and subsequent chitosan coating were achieved through electrostatic interactions, ensuring that both the drug-loading and the drug-releasing processes are well-controlled. In vitro drug-release experiments revealed a noticeable responsiveness of the drug carrier upon triggering by both the pH and the peptide glutathione (the drug was completely released within 54 h). Concerning the prevention and control of electromagnetic waves, carbon-based hollow spherical shells have attracted much attention in the recent decades. In [14] the investigated carbon hollow microspheres, with the apparent benefit of low density and also of structural integrity, exhibited efficient absorption of electromagnetic waves due to both interfacial polarization and multireflection. Furthermore, those spherical units serve as the parent structure for the development of mesoporous alternates which exhibit additional advantages. Such mesoporous carbon hollow microspheres were studied in [15] exhibiting tailored textural and dielectric properties that enable the absorption of electromagnetic waves very effectively. In [16] the dielectric hollow spherical shells (SiC) were upgraded with the addition of magnetic ingredients (Ni), thus exhibiting improved performance on the absorption of electromagnetic waves, in comparison to simple dielectric shells. In [17] systematic series of carbon hollow microspheres were synthesized from phenolic resin coated on polystyrene microspheres. These spherical units were highly monodisperse in respect to their structure (size and thickness) and physical properties (microwave absorption). Except for the intrinsic mechanism of dipole polarization relaxation, the overall dielectric loss of those spherical units is influenced by the extrinsic mechanism of the interfacial polarization due to their heterogeneous interface with air. In [18] hollow microspheres were synthesized as dual-shells of Carbon and Fe, thus combining the dielectric and magnetic properties, as efficient microwave absorbers over a broad bandwidth. Such lightweight and structurally stable spherical units are appropriate for electronic, aerospace and defense applications.

A common trend of the last decades is the size reduction in such dielectric and magnetic building units, toward the achievement of a high surface area/volume ratio. In most cases this property facilitates applications [19,20,21,22,23,24,25]. However, this advantage comes at the expense of the appearance of physical mechanisms that can exert an antagonistic action to the desired applications, e.g., engineering of an external potential at both the inside and outside spaces of the hollow shell. Such a physical mechanism, inherent to both dielectric and magnetic building units, is the so-called depolarization effect. This effect can impose serious technical drawbacks that, if not resolved, can become critically detrimental to applications. Indeed, in cases of significant size reduction the surface of a building unit is in close vicinity to the whole of its volume. Accordingly, any physical mechanism that stems from the surface can strongly influence physical processes that take place in the volume of the building unit. The depolarization effect is probably the most well-known physical mechanism of this origin since it always stems from the surface of a building unit, if not even from its volume (see [19,26,27,28,29] for the dielectric case and [23,27,30,31,32] for the magnetic case). In macro/meso-scopic three-dimensional specimens, of low surface area/volume ratio, the depolarization effect is practically negligible, while in micro/nano-scopic two/one-dimensional building units it even can dominate their response to an external trigger. Indeed, in applications an external electric/magnetic field, $E_{ext}(r)/H_{ext}(r)$, produced by primary/free sources placed outside a building unit, will induce an electric/magnetic polarization, P(r)/M(r), in its entire volume. The polarization always exhibits a discontinuity at the surface of such building units so that secondary/bound sources are always induced at the interface with the outside space. These induced sources will produce a secondary electric/magnetic field, the so-called internal field, $E_{int}(r)/H_{int}(r)$, that actually is the response of the building unit to the external trigger [33,34,35,36,37,38]. Due to the high surface area/volume ratio of the building unit and the relatively long range of action of the internal electric/magnetic field, $E_{int}(r)/H_{int}(r)$, the latter will influence the entire volume of the building unit. It should be noted that due to its physical origin the internal field, $E_{int}(r)/H_{int}(r)$, always opposes to the external one, $E_{ext}(r)/H_{ext}(r)$, tending to eliminate it from the interior of the building unit (shielding effect). Thus, the internal field, $E_{int}(r)/H_{int}(r)$, actually tends to depolarize the building unit. For that reason, the process is termed depolarization effect and the above-mentioned internal field, $E_{int}(r)/H_{int}(r)$, is equivalently termed depolarization field, $E_{d}(r)/H_{d}(r)$ or self-field, $E_{s}(r)/H_{s}(r)$ [33,34,35,36,37,38].

**Figure 1:**
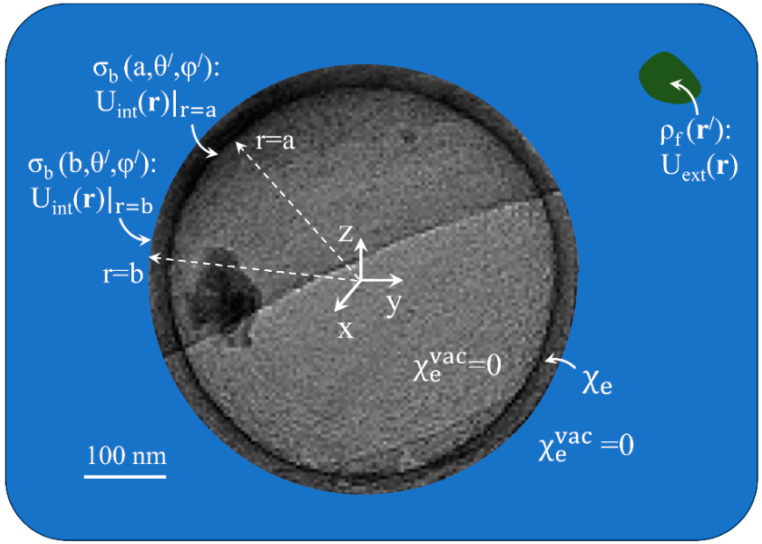
Schematic illustration based on a transmission electron microscopy image of a carbon hollow microsphere [14] (reproduced by permission). A homogeneous dielectric hollow spherical shell of constant intrinsic susceptibility, $\chi_{e}$, is subjected to an external electric potential, $U_{ext}\left(r\right)$, of *any* form (dc or ac of low frequency), originating from a primary/free volume source, $\rho_{f}(r^{/})$, that resides outside the spherical hollow shell. Two secondary/bound surface sources, $\sigma_{b}(b,\theta^{/},\varphi^{/})$ and $\sigma_{b}(a,\theta^{/},\varphi^{/})$, are established at the outer and inner surface, r=b and r=a, respectively, producing the relevant components of the internal potential, $U_{int}\left(r\right){|}_{r=b}$ and $U_{int}\left(r\right){|}_{r=a}$. The total internal potential is $U_{int}\left(r\right)=U_{int}\left(r\right){|}_{r=b}+U_{int}\left(r\right){|}_{r=a}$. See text for details.

The exploration of the depolarization effect is most commonly limited to the rectangular geometry of a plate since this is usually met in a plethora of applications based on films [30]. Clearly, dielectric and magnetic spherical structures have not been investigated to an analogous extent [39,40,41,42,43]. Even when such structures are considered, they are always subjected to a homogeneous external field. Only recently, the investigation of the depolarization effect met in solid dielectric and magnetic spheres and cylinders, consisting of a linear, homogeneous and isotropic parent material, was expanded to inhomogeneous external fields of *any* spatial form [44,45,46]. However, the case of linear, homogeneous and isotropic spherical hollow shells has not been addressed adequately, neither analytically nor computationally, in the literature [47,48,49]. Even when such hollow structures are considered, they are always subjected to a homogeneous external field, just like their solid counterparts [47,48,49]. Here we employ standard electromagnetism to tackle the problem of such dielectric and magnetic spherical hollow shells of inner and outer radius, a and b, respectively. In both cases these structures are subjected to an external potential, $U_{ext}\left(r\right)$, of *any* spatial form (either dc (static) or ac of low-frequency (quasistatic limit)) produced by primary/free sources that reside at the outside space, Figure 1. In this case the depolarization effect is enhanced due to the existence of two surfaces (r=a and r=b), instead of the one (r=b) that exists in the solid spheres. We apply the method-of-linear-recursive-solution (MLRS) to the Laplace equation, an expansion-based mathematical procedure introduced in [44,45,46], and calculate the internal and total potential, $U_{int}\left(r\right)$ and $U_{tot}\left(r\right)$, everywhere in space. From the analytical expressions obtained by the MLRS we calculate all relevant internal and total scalar and vector physical entities of interest. An apparent advantage of the MLRS is that it enables us to obtain closed-form expressions for the depolarization factor that emerge at both the outer (r=b) and the inner (r=a) surface of such dielectric and magnetic spherical hollow shells. Our results are directly relevant to several application-driven settings treated in detail in this work. First, we apply the framework to resonance frequencies in scattering problems, using the model to estimate the plasma frequency under resonance conditions. Second, we address materials characterization in magnetometry by performing detailed comparisons with previously published experimental data to extract the intrinsic magnetic susceptibility from measured responses. Third, we study dielectric shielding and distortion, deriving analytical expressions and supporting simulations. This analysis also clarifies that varying the inner and outer radii offers limited geometric leverage for controlling the shielding at the inside space relatively to the distortion at the outside space. More broadly, the generic MLRS framework—implemented either analytically or computationally—enables the systematic understanding and tailoring of depolarization processes in other dielectric and magnetic building-unit geometries encountered in applications.

## 2. Background of the Method-of-Linear-Recursive-Solution (MLRS) of the Laplace Equation and Connection to the Physical Problem

In this section we first define the physical problem following the MLRS of the Laplace equation introduced in [44,45,46] for the cases of solid spheres and cylinders consisting of linear, homogeneous and isotropic dielectric and magnetic materials. Actually, in all applications discussed above the external potential, $U_{ext}\left(r\right)$, (dc or ac of low frequency) which triggers the response, $U_{int}\left(r\right)$, of the building unit stems from primary/free sources that are placed in the outside space. Figure 1 shows an illustration of a dielectric spherical hollow shell, of inner and outer radius, a and b, respectively, studied here that consists of linear, homogeneous and isotropic material of constant intrinsic susceptibility, $\chi_{e}$. The illustration is based on a TEM image of a carbon hollow microsphere [14]. The primary source of free charges, $\rho_{f}(r)$, resides outside the spherical building unit. Secondary sources of bound charges, $\sigma_{b}\left(r\right){|}_{S}=P(r)\cdot \hat{n}{|}_{S}$, are induced at both the inner and outer surfaces, r=a and r=b, respectively, of the spherical hollow shell due to the discontinuity of the electric polarization, P(r). The homogeneous character of the dielectric material ensures that no volume source of bound charges exists, $\rho_{b}\left(r\right)=-\nabla \cdot P\left(r\right)=0$. The secondary/bound sources, $\sigma_{b}\left(r\right){|}_{S}=P(r)\cdot \hat{n}{|}_{S}$, produce the response of the dielectric spherical hollow shell, that is the internal electric potential, $U_{int}\left(r\right)$. The main goal of our work is to calculate $U_{int}\left(r\right)$.

The scalar potential, U(r), is the physical entity of paramount importance for all theoretical and experimental investigations since it is the parent scalar field of all scalar and vector physical entities that appear in relevant problems. Electric/magnetic field, E(r)/H(r); polarization, P(r); bound charge/pseudocharge densities, volume, ρ_b_(**r**); surface, $\sigma_{b}(r){|}_{s}$; ones, electric/magnetic moment, p/m; force, F; torque, T; etc. can be obtained analytically when the scalar potential, U(r), is known everywhere in space [33,34,35,36,37,38]. Going a step further, when referring to experiments and applications, what is actually important is the response of the studied building unit of known characteristics (i.e., shape, dimensions and intrinsic electric/magnetic susceptibility, $\chi_{e}/\chi_{m}$) to an external trigger of known form, i.e., external scalar potential, $U_{ext}\left(r\right)$, of known spatial distribution. Accordingly, we should be able to calculate the internal scalar potential, $U_{int}\left(r\right)$, of the building unit as function of the above-mentioned known parameters. Then, the total scalar potential should be known, as well, thanks to the superposition principle, $U_{tot}\left(r\right)=U_{ext}\left(r\right)+U_{int}\left(r\right)$.

To this end, here we employ the MLRS of the Laplace equation developed in [44,45,46] for the investigation of solid spheres and cylinders consisting of linear, homogeneous and isotropic dielectric and magnetic materials. Originally, the MLRS was based on the construction of a non-local, integro-differential equation for the polarization of a solid sphere subjected to an external electric/magnetic potential of any form [44]. In [45,46] it was updated for the direct calculation of the internal and total electric/magnetic field, instead of the electric/magnetic polarization. Here, we employ the MLRS in a more compact and rather more simple form to directly calculate the internal and total electric/magnetic potential of spherical hollow shells.

**Figure 2:**
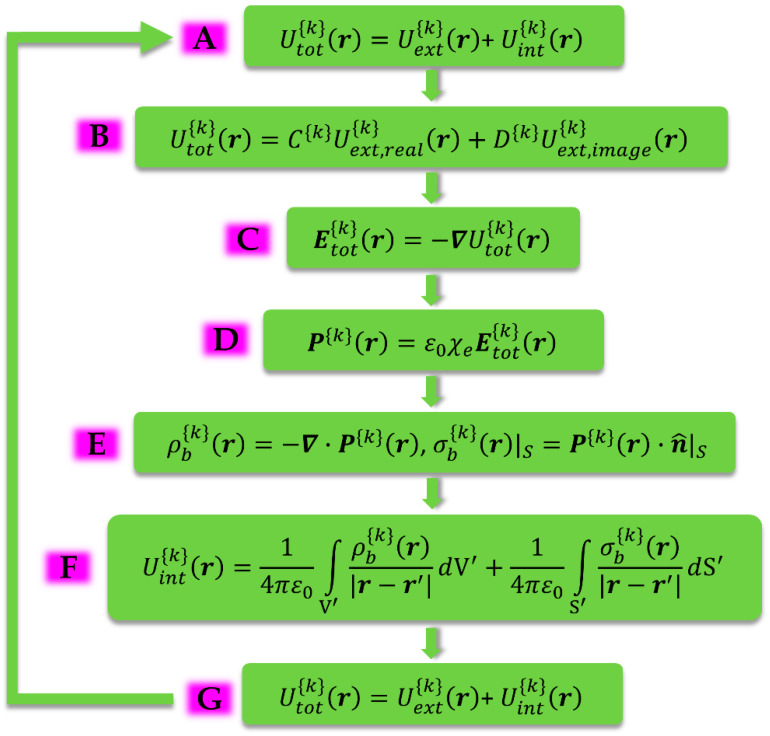
Block diagram that summarizes the basic steps, A to G, of the method-of-linear-recursive-solution (MLRS) employed to address the Laplace equation within the volume of a linear, homogeneous and isotropic dielectric and magnetic material. MLRS is applied here to the dielectric case, to calculate the internal potential, $U_{int}\left(r\right)$, within the volume of a spherical hollow shell of intrinsic susceptibility, $\chi_{e}$, subjected to a dc (static) or ac of low-frequency (quasistatic limit) external potential, $U_{ext}\left(r\right)$, of any spatial form. Thus, the diagram refers to the dielectric case. The magnetic one follows the exact same argumentation. In the diagram only the general term of each physical entity of interest is shown. The complete result for each case is obtained by the summation over all valid (i.e., non-zero) modes {k} on the basis of the respective space (e.g., {k}=(l,m) for the spherical case studied here where the SH, $Y_{l}^{m}(\theta ,\varphi )$, is the relevant basis). See text for details.

The mathematical workflow is shown schematically in Figure 2, below. We recall that the mathematical part relies on a series-based expansion on the basis of the respective spherical and cylindrical space [44,45,46]. For convenience, in the block diagram of Figure 2 we refer only to the general term of the respective series that describes each physical entity. Generalization to the entire physical entities is easily obtained through a simple summation over all existing (i.e., non-zero) modes {k}. For instance, for the case of spherical hollow shells the relevant basis is the spherical harmonics (SH), $Y_{l}^{m}(\theta ,\varphi )$, so that the modes {k} refer to {k}=(l,m), while for the case of cylindrical hollow shells they are the so-called Cylindrical Harmonics [50]. The MLRS employed for the calculation of the internal and total electric potentials, $U_{int}\left(r\right)$ and $U_{tot}\left(r\right)$, presented in Figure 2, starts at step A and ends at step G with the superposition principle; the comparison of the solution employed in step A with the one obtained in step G should give a self-consistent result. To proceed in step B, a constitutive relation should be employed between the external trigger, $U_{ext}\left(r\right)$, and the internal response, $U_{int}\left(r\right)$. Our choice is based on a simple fact that practically is always disregarded in electromagnetic theory: a specimen constituting of a linear, homogeneous and isotropic dielectric/magnetic material should respond linearly to the external trigger, i.e., to the external electric potential, $U_{ext}\left(r\right)$, produced by the primary/free sources. This means that the response of the specimen, i.e., the internal electric/magnetic potential, $U_{int}\left(r\right)$, produced by the secondary/bound sources, should be linear to $U_{ext}\left(r\right)$. Thus, the following simple relation should hold, $U_{int}\left(r\right)\propto U_{ext}\left(r\right)$, so that the total electric potential, $U_{tot}\left(r\right)$, should exhibit an analogous linear behavior, $U_{tot}\left(r\right)\propto U_{ext}\left(r\right)$, as well. On physical grounds, the general term of the internal potential, $U_{int}^{\left\{k\right\}}\left(r\right)$ (step A), can be considered as an *image* external source, $U_{ext,image}^{\left\{k\right\}}\left(r\right)$, originating from the *real* external source (step B), as usually assumed in more simple cases where primary/free sources (e.g., a free point electric charge/magnetic pseudocharge) are placed outside magnetic/dielectric spheres [51,52,53,54] (details will be discussed elsewhere). Accordingly, the general term of the total potential, $U_{tot}^{\left\{k\right\}}\left(r\right)$, can be considered as the sum of the respective general terms, $U_{ext,real}^{\left\{k\right\}}\left(r\right)$, and $U_{ext,image}^{\left\{k\right\}}\left(r\right)$. A crucial point that should be highlighted is that the terms $U_{ext,real}^{\left\{k\right\}}\left(r\right)$ and $U_{ext,image}^{\left\{k\right\}}\left(r\right)$ contribute to $U_{tot}^{\left\{k\right\}}\left(r\right)$ through different linearity coefficients, $C^{\left\{k\right\}}$ and $D^{\left\{k\right\}}$, respectively (step B). Once this point is clarified the rest of the algebra is relatively simple. In step C the general term of the total electric field, $E_{tot}^{\left\{k\right\}}\left(r\right)$, is calculated from $U_{tot}^{\left\{k\right\}}\left(r\right)$. In step D the general term of the polarization, $P^{\left\{k\right\}}\left(r\right)$, is also directly obtained from $E_{tot}^{\left\{k\right\}}\left(r\right)$ and the known intrinsic susceptibility, $\chi_{e}$, of the dielectric material. In step E the general terms of the secondary/bound sources are calculated from $P^{\left\{k\right\}}\left(r\right)$. These always include surface ones, $\sigma_{b}^{\left\{k\right\}}\left(r\right)$, that surely exist at both surfaces, r=a (inner) and r=b (outer), of the spherical hollow shell due to the discontinuity of $P^{\left\{k\right\}}\left(r\right)$. In addition, volume ones, $\rho_{b}^{\left\{k\right\}}\left(r\right)$, can probably exist at the interior of the spherical hollow shell. However, in the cases studied here $\rho_{b}^{\left\{k\right\}}\left(r\right)=0$ always holds due to the linear, homogeneous and isotropic character of the dielectric/magnetic material resulting in the divergence-free character of $P^{\left\{k\right\}}\left(r\right)$. In step F the general term of the internal potential, $U_{int}^{\left\{k\right\}}\left(r\right)$, is immediately calculated from the secondary/bound sources, $\sigma_{b}^{\left\{k\right\}}\left(r\right)$, by using the generalized law of Coulomb, aided by a series-based expansion (i.e., the multipole one). Then, in step G the general term of the total electric potential, $U_{tot}^{\left\{k\right\}}\left(r\right)$, is recovered from $U_{int}^{\left\{k\right\}}\left(r\right)$, once again thanks to the superposition principle. The expression of $U_{tot}^{\left\{k\right\}}\left(r\right)$ obtained in the final step G should be identical to the one obtained in the initial step A. The requirement of a self-consistent solution of $U_{tot}^{\left\{k\right\}}\left(r\right)$ enables us to calculate the linearity coefficients, $C^{\left\{k\right\}}$ and $D^{\left\{k\right\}}$. These ultimately relate to the depolarization factors that originate from the inner, r=a, and outer, r=b, surfaces of the spherical hollow shell, as shown below.

The MLRS of the Laplace equation described above is employed in the dielectric and magnetic spherical hollow shells discussed in the following sections of our work. In Section 3 we apply the method in detail to a dielectric spherical hollow shell. In Section 4 we summarize briefly the respective results for the magnetic counterpart.

## 3. Dielectric Spherical Hollow Shells That Consist of a Linear, Homogeneous and Isotropic Material

We start our discussion with the expansion of the external electric potential, $U_{ext}\left(r\right)$, on the basis of SH [44,45] that are defined through the relation

(1) $$Y_{l}^{m}\left(\theta ,\varphi \right)=\sqrt{\frac{2l+1}{4\pi }\frac{\left(l-m\right)!}{\left(l+m\right)!}}P_{l}^{m}\left(cos\theta \right)e^{im\varphi }$$

and are subjected to the orthonormality condition

(2) $$\int_{0}^{2\pi }\int_{0}^{\;\pi }Y_{l^{\prime }}^{m^{\prime }}\left(\theta^{\prime },\varphi^{\prime }\right)Y_{l}^{m*}\left(\theta^{\prime },\varphi^{\prime }\right)sin\theta^{\prime }d\theta^{\prime }d\varphi^{\prime }=\delta_{ll^{\prime }}\delta_{{mm}^{\prime }}$$

In general, $U_{ext}\left(r\right)$ can have the following form:

(3) $$U_{ext}\left(r\right)=\sum_{l=0}^{\infty }\sum_{m=-l}^{l}\left(A_{ext}^{\left(l,m\right)}r^{l}+B_{ext}^{\left(l,m\right)}r^{-\left(l+1\right)})Y_{l}^{m}(\theta ,\varphi \right)$$

However, in the applications discussed above the primary/free source is placed outside the spherical hollow shell. Thus, in what follows we always refer to the inside space of the primary/free source so that the term $B_{ext}^{\left(l,m\right)}r^{-\left(l+1\right)}$ should be omitted. Accordingly, $B_{ext}^{\left(l,m\right)}=0$ for all modes (l,m). Thus, the external scalar potential is given by the relation

(4) $$U_{ext}\left(r\right)=\sum_{l=0}^{\infty }\sum_{m=-l}^{l}U_{ext}^{\left(l,m\right)}\left(r\right)=\sum_{l=0}^{\infty }\sum_{m=-l}^{l}A_{ext}^{\left(l,m\right)}r^{l}Y_{l}^{m}(\theta ,\varphi )$$

where we have defined the general term of the expansion through

(5) $$U_{ext}^{\left(l,m\right)}\left(r\right)=A_{ext}^{\left(l,m\right)}r^{l}Y_{l}^{m}(\theta ,\varphi )$$

The expansion coefficients $A_{ext}^{\left(l,m\right)}$ are obtained through the relation

(6) $$A_{ext}^{\left(l,m\right)}=\frac{1}{r^{l}}\int_{0}^{2\pi }\int_{0}^{\pi }U_{ext}\left(r\right)Y_{l}^{m*}\left(\theta ,\varphi \right)sin\theta d\theta d\varphi$$

In this relation $U_{ext}\left(r\right)$ obeys the separation of variables, $U_{ext}\left(r\right)=U_{0}U_{ext}\left(r\right)U_{ext}\left(\theta \right)U_{ext}\left(\varphi \right)$, so that its radial component, $U_{ext}\left(r\right)$, can be brought out of the integral and eliminate the term $1/r^{l}$, thus recovering the constant character of the expansion coefficients, $A_{ext}^{\left(l,m\right)}$. Relations (4)–(6) actually determine the values of the degree, l, and order, m, of the existing modes (l,m). It should be noted that the above expression of $U_{ext}\left(r\right)$ holds in the entire space of interest that is outside ($b\leq r\leq r_{fs}$), middle (a≤r≤b) and inside (0≤r≤a) of the spherical hollow shell (where $r_{fs}$ stands for the position of the primary source of free charges).

### 3.1. Dielectric Spherical Hollow Shell: Middle Space (a≤r≤b)

First, we focus onto the middle space (a≤r≤b) wherein we apply analytically the MLRS to the Laplace equation since the polarization is non-zero only in this regime. To calculate the total potential, $U_{tot}\left(r\right)$, we still need an expression for the internal potential, $U_{int}\left(r\right)$, produced by the secondary sources of the bound charges that reside at the inner and outer surfaces, r=a and r=b, respectively. Once in the middle space $U_{int}\left(r\right)$ is always finite, the following general form should be used

(7) $$U_{int}\left(r\right)=\sum_{l=0}^{\infty }\sum_{m=-l}^{l}\left(A_{mid}^{\left(l,m\right)}r^{l}+B_{mid}^{\left(l,m\right)}r^{-\left(l+1\right)}\right)Y_{l}^{m}(\theta ,\varphi )$$

Accordingly, the total potential, $U_{tot}\left(r\right)=U_{ext}\left(r\right)+U_{int}\left(r\right)$, is given by the expression

(8) $$U_{tot}\left(r\right)=\sum_{l=0}^{\infty }\sum_{m=-l}^{l}\left(A_{ext}^{\left(l,m\right)}+A_{mid}^{\left(l,m\right)}\right)r^{l}Y_{l}^{m}(\theta ,\varphi )+\sum_{l=0}^{\infty }\sum_{m=-l}^{l}B_{mid}^{\left(l,m\right)}r^{-\left(l+1\right)}Y_{l}^{m}(\theta ,\varphi )$$

The first term is the potential produced in the middle space by the primary source of free charges ($\rho_{f}(r)$) and the secondary source of bound charges ($\sigma_{b}(b,\theta^{/},\varphi^{/})$) that resides at the outer surface, r=b, of the spherical hollow shell. Thus, the first term is actually equal to $U_{ext}\left(r\right)+U_{int}\left(r\right){|}_{r=b}$. The second term is the potential produced in the middle space by the secondary source of bound charges ($\sigma_{b}(a,\theta^{/},\varphi^{/})$) that reside at the inner surface, r=a, of the spherical hollow shell. Thus, the second term is actually $U_{int}\left(r\right){|}_{r=a}$. Going a step farther, the second term can be considered as an extra primary source produced by the image of the free charges at the inside space of the spherical hollow shell [51,52,53,54] (details will be discussed elsewhere).

Once we have concluded with step A of Figure 2 we can proceed to step B by using the above relation (8) and imposing the linearity between $U_{tot}\left(r\right)$ and $U_{ext}\left(r\right)$, for each one of the two terms ($r^{l}$ and $r^{-\left(l+1\right)}$), thus obtaining the following relation:

(9) $$U_{tot}\left(r\right)=\sum_{l=0}^{\infty }\sum_{m=-l}^{l}C^{\left(l,m\right)}A_{ext}^{\left(l,m\right)}r^{l}Y_{l}^{m}(\theta ,\varphi )+\sum_{l=0}^{\infty }\sum_{m=-l}^{l}D^{\left(l,m\right)}r^{-\left(l+1\right)}Y_{l}^{m}(\theta ,\varphi )$$

Going to the next step C of Figure 2 we calculate the total field, $E_{tot}\left(r\right)$, from the total potential, $U_{tot}\left(r\right)$, through the standard relation E(r)=−∇U(r), by using the above relation (9). Thus, we obtain

(10) $$E_{tot}\left(r\right)=-\sum_{l=0}^{\infty }\sum_{m=-l}^{l}C^{\left(l,m\right)}A_{ext}^{\left(l,m\right)}\nabla \left(r^{l}Y_{l}^{m}\left(\theta ,\varphi \right)\right)-\sum_{l=0}^{\infty }\sum_{m=-l}^{l}D^{\left(l,m\right)}\nabla \left(r^{-\left(l+1\right)}Y_{l}^{m}\left(\theta ,\varphi \right)\right)$$

Next, we proceed to step D of Figure 2 and directly obtain the electric polarization of the middle space, through the constitutive relation $P\left(r\right)=\varepsilon_{0}\chi_{e}E_{tot}\left(r\right)$, by using the above relation (10). We easily obtain

(11) $$P\left(r\right)=-\varepsilon_{0}\chi_{e}\sum_{l=0}^{\infty }\sum_{m=-l}^{l}C^{\left(l,m\right)}A_{ext}^{\left(l,m\right)}\nabla \left(r^{l}Y_{l}^{m}\left(\theta ,\varphi \right)\right)-\varepsilon_{0}\chi_{e}\sum_{l=0}^{\infty }\sum_{m=-l}^{l}D^{\left(l,m\right)}\nabla \left(r^{-\left(l+1\right)}Y_{l}^{m}\left(\theta ,\varphi \right)\right)$$

Following step E of Figure 2 we calculate the two secondary sources of bound charges that reside at the two surfaces of the spherical hollow shell, the inner (r=a) and the outer (r=b). We use the standard definition $\sigma_{b}\left(r\right){|}_{S}=P(r)\cdot \hat{n}{|}_{S}$ and the above relation (11). By recalling that $\hat{n}=-\hat{r}$ at the inner surface (r=a), while $\hat{n}=\hat{r}$ at the outer surface (r=b), after some simple algebra we obtain

(12) $$\sigma_{b}\left(r\right){|}_{r=a}=\varepsilon_{0}\chi_{e}\sum_{l=0}^{\infty }\sum_{m=-l}^{l}C^{\left(l,m\right)}A_{ext}^{\left(l,m\right)}la^{l-1}Y_{l}^{m}\left(\theta ,\varphi \right)-\varepsilon_{0}\chi_{e}\sum_{l=0}^{\infty }\sum_{m=-l}^{l}D^{\left(l,m\right)}\left(l+1\right)a^{-\left(l+2\right)}Y_{l}^{m}\left(\theta ,\varphi \right)$$

and

(13) $$\sigma \left(r\right){|}_{r=b}=-\varepsilon_{0}\chi_{e}\sum_{l=0}^{\infty }\sum_{m=-l}^{l}C^{\left(l,m\right)}A_{ext}^{\left(l,m\right)}lb^{l-1}Y_{l}^{m}\left(\theta ,\varphi \right)+\varepsilon_{0}\chi_{e}\sum_{l=0}^{\infty }\sum_{m=-l}^{l}D^{\left(l,m\right)}\left(l+1\right)b^{-\left(l+2\right)}Y_{l}^{m}\left(\theta ,\varphi \right)$$

From these relations we clearly see that the bound charges, $\sigma_{b}\left(r\right){|}_{r=a}$ and $\sigma_{b}\left(r\right){|}_{r=b}$, induced at the inner, r=a, and outer, r=b, surface are opposite, as expected. *Accordingly, these bound charges of the inner- and outer-facing surfaces are coupled, thus producing an internal field in the middle space that tends to depolarize the material. This is why the internal field is also termed depolarization field (see below).*

Now we can proceed to step F of Figure 2 and calculate the two components of the internal potential, $U_{int}\left(r\right)$, produced by the respective bound charges by using the generalized law of Coulomb [33,34,37,38]. The one that stems from $\sigma_{b}\left(r\right){|}_{r=a}$ is termed $U_{int}\left(r\right){|}_{r=a}$, while the one that stems from $\sigma_{b}\left(r\right){|}_{r=b}$ is termed $U_{int}\left(r\right){|}_{r=b}$. To this end we need the expansion of ${\left|r-r^{\prime }\right|}^{-1}$ on the basis of SH for the inside and outside spaces ($r<r^{\prime }$ and $r^{\prime }<r$, respectively) given by [44,45]

(14) $$\frac{1}{\left|r-r^{\prime }\right|}=4\pi \sum_{l=0}^{\infty }\sum_{m=-l}^{l}\frac{1}{2l+1}\frac{\{r^{l},{r^{\prime }}^{l}{\}}_{<}}{\{r^{l+1},{r^{\prime }}^{l+1}{\}}_{>}}Y_{l}^{m}(\theta ,\varphi )Y_{l}^{m*}\left(\theta^{\prime },\varphi^{\prime }\right)$$

where $\{r^{l},{r^{\prime }}^{l}{\}}_{<}$ and $\{r^{l+1},{r^{\prime }}^{l+1}{\}}_{>}$ denote the lower and higher, in magnitude, between the two vectors, r and $r^{\prime }$ (which run over the volume of observation and sources, respectively). Thus, for the inside space ($r<r^{\prime }$), $\{r^{l},{r^{\prime }}^{l}{\}}_{<}=r^{l}$ and $\{r^{l+1},{r^{\prime }}^{l+1}{\}}_{>}={r^{\prime }}^{l+1}$, while for the outside space $\left(r^{\prime }<r\right)$, $\{r^{l},{r^{\prime }}^{l}{\}}_{<}={r^{\prime }}^{l}$ and $\{r^{l+1},{r^{\prime }}^{l+1}{\}}_{>}=r^{l+1}$.

To calculate $U_{int}\left(r\right){|}_{r=a}$ we need $\sigma_{b}\left(r\right){|}_{r=a}$ from relation (12) and the expansion of the outside space from relation (14). Inserting this information into the generalized law of Coulomb, after some algebra we obtain

(15) $$U_{int}\left(r\right){|}_{r=a}=\chi_{e}\sum_{l^{\prime }=0}^{\infty }\sum_{m^{\prime }=-l^{\prime }}^{l^{\prime }}C^{\left(l^{\prime },m^{\prime }\right)}A_{ext}^{\left(l^{\prime },m^{\prime }\right)}l^{\prime }a^{l^{\prime }-1}\sum_{l=0}^{\infty }\sum_{m=-l}^{l}\frac{1}{2l+1}\frac{a^{l+2}}{r^{l+1}}Y_{l}^{m}\left(\theta ,\varphi \right)\int_{0}^{2\pi }\int_{0}^{\pi }Y_{l^{\prime }}^{m^{\prime }}\left(\theta^{\prime },\varphi^{\prime }\right)Y_{l}^{m*}\left(\theta^{\prime },\varphi^{\prime }\right)sin\theta^{\prime }d\theta^{\prime }d\varphi^{\prime }\;-\chi_{e}\sum_{l^{\prime }=0}^{\infty }\sum_{m^{\prime }=-l^{\prime }}^{l^{\prime }}D^{\left(l^{\prime },m^{\prime }\right)}(l^{\prime }+1)a^{-(l^{\prime }+2)}\sum_{l=0}^{\infty }\sum_{m=-l}^{l}\frac{1}{2l+1}\frac{a^{l+2}}{r^{l+1}}Y_{l}^{m}\left(\theta ,\varphi \right)\int_{0}^{2\pi }\int_{0}^{\pi }Y_{l^{\prime }}^{m^{\prime }}\left(\theta^{\prime },\varphi^{\prime }\right)Y_{l}^{m*}\left(\theta^{\prime },\varphi^{\prime }\right)sin\theta^{\prime }d\theta^{\prime }d\varphi^{\prime }$$

To calculate $U_{int}\left(r\right){|}_{r=b}$ we need $\sigma_{b}\left(r\right){|}_{r=b}$ from relation (13) and the expansion of the inside space from relation (14). Inserting this information into the generalized law of Coulomb, after some algebra we obtain

(16) $$U_{int}\left(r\right){|}_{r=b}=-\chi_{e}\sum_{l^{\prime }=0}^{\infty }\sum_{m^{\prime }=-l^{\prime }}^{l^{\prime }}C^{\left(l^{\prime },m^{\prime }\right)}A_{ext}^{\left(l^{\prime },m^{\prime }\right)}l^{\prime }b^{l^{\prime }+1}\sum_{l=0}^{\infty }\sum_{m=-l}^{l}\frac{1}{2l+1}\frac{r^{l}}{b^{l+1}}Y_{l}^{m}\left(\theta ,\varphi \right)\int_{0}^{2\pi }\int_{0}^{\pi }Y_{l^{\prime }}^{m^{\prime }}\left(\theta^{\prime },\varphi^{\prime }\right)Y_{l}^{m*}\left(\theta^{\prime },\varphi^{\prime }\right)sin\theta^{\prime }d\theta^{\prime }d\varphi^{\prime }\;+\chi_{e}\sum_{l^{\prime }=0}^{\infty }\sum_{m^{\prime }=-l^{\prime }}^{l^{\prime }}D^{\left(l^{\prime },m^{\prime }\right)}\left(l^{\prime }+1\right)b^{-l^{\prime }}\sum_{l=0}^{\infty }\sum_{m=-l}^{l}\frac{1}{2l+1}\frac{r^{l}}{b^{l+1}}Y_{l}^{m}\left(\theta ,\varphi \right)\int_{0}^{2\pi }\int_{0}^{\pi }Y_{l^{\prime }}^{m^{\prime }}\left(\theta^{\prime },\varphi^{\prime }\right)Y_{l}^{m*}\left(\theta^{\prime },\varphi^{\prime }\right)sin\theta^{\prime }d\theta^{\prime }d\varphi^{\prime }$$

In the above expressions the integrals are actually the orthonormality relation (2) so that we easily obtain the more convenient forms

(17) $$U_{int}\left(r\right){|}_{r=a}=\chi_{e}\sum_{l^{\prime }=0}^{\infty }\sum_{m^{\prime }=-l^{\prime }}^{l^{\prime }}\left(C^{\left(l^{\prime },m^{\prime }\right)}A_{ext}^{\left(l^{\prime },m^{\prime }\right)}\frac{l^{\prime }}{2l^{\prime }+1}a^{2l^{\prime }+1}-D^{\left(l^{\prime },m^{\prime }\right)}\frac{l^{\prime }+1}{2l^{\prime }+1}\right)\frac{Y_{l^{\prime }}^{m^{\prime }}\left(\theta ,\varphi \right)}{r^{l^{\prime }+1}}$$

and

(18) $$U_{int}\left(r\right){|}_{r=b}=-\chi_{e}\sum_{l^{\prime }=0}^{\infty }\sum_{m^{\prime }=-l^{\prime }}^{l^{\prime }}\left(C^{\left(l^{\prime },m^{\prime }\right)}A_{ext}^{\left(l^{\prime },m^{\prime }\right)}\frac{l^{\prime }}{2l^{\prime }+1}-D^{\left(l^{\prime },m^{\prime }\right)}\frac{l^{\prime }+1}{2l^{\prime }+1}\frac{1}{b^{2l^{\prime }+1}}\right)r^{l^{\prime }}Y_{l^{\prime }}^{m^{\prime }}\left(\theta ,\varphi \right)$$

Accordingly, by rejecting the prime notation, the internal potential of the middle space (a≤r≤b), $U_{int}\left(r\right)=U_{int}\left(r\right){|}_{r=a}+U_{int}\left(r\right){|}_{r=b}$, is given by the following relation

(19) $$U_{int}\left(r\right)=-\chi_{e}\sum_{l=0}^{\infty }\sum_{m=-l}^{l}\left(C^{\left(l,m\right)}A_{ext}^{\left(l,m\right)}\frac{l}{2l+1}\left(r^{l}-\frac{a^{2l+1}}{r^{l+1}}\right)+D^{\left(l,m\right)}\frac{l+1}{2l+1}\left(-\frac{r^{l}}{b^{2l+1}}+\frac{1}{r^{l+1}}\right)\right)Y_{l}^{m}\left(\theta ,\varphi \right)$$

Previous investigations of ours referred to solid spheres consisting of linear, homogeneous and isotropic dielectric material [44,45]. In those works, we defined the depolarization factor, $N_{l}$, and the extrinsic electric susceptibility, $\chi_{e,l}^{ext}$, of degree, l, that are valid for any form of the external electric potential, $U_{ext}\left(r\right)$, by the relations

(20) $$N_{l}=\frac{l}{2l+1}$$

and

(21) $$\chi_{e,l}^{ext}=\frac{\chi_{e}^{int}}{1+N_{l}\chi_{e}^{int}}$$

where $\chi_{e}^{int}$ is the intrinsic electric susceptibility of the material that refers to its truly endogenous properties (simply $\chi_{e}$ in the notation of the present work). Those results [44,45] evidenced that both $N_{l}$ and $\chi_{e,l}^{ext}$ are degenerate over the 2l+1 available values of the order, m (−l≤m≤l).

In the above relation (19), obtained here for a spherical hollow shell subjected to an external electric potential of any form, we see that the depolarization factor, $N_{l}$, appears in the first term. Interestingly, another similar factor appears in the second term so that we define another depolarization factor of degree l+1, $N_{l+1}$, through the relation

(22) $$N_{l+1}=\frac{l+1}{2l+1}$$

*The above clearcut results compel us to ascribe the depolarization factor,* $N_{l}$*, of degree,* l*, to the outer surface,* r=b*, and the depolarization factor,* $N_{l+1}$*, of degree,* l+1*, to the inner surface,* r=a*, of the spherical hollow shell. These two depolarization factors,* $N_{l}$ *and* $N_{l+1}$*, depend on the degree,* l*, of the mode of the external potential,* $U_{ext}^{\left(l,m\right)}\left(r\right)$*. However,* $N_{l}$*, and,* $N_{l+1}$*, are independent from the order,* m*, thus being degenerate over its,* 2l+1*, values,* −l≤m≤l*. Importantly, the sum of these two distinct depolarization factors equals unity,* $N_{l}+N_{l+1}=1$*, independently of the degree,* l*, of the mode of the external potential,* $U_{ext}^{\left(l,m\right)}\left(r\right)$*, as it should.*

Using the definitions of relations (20) and (22), the internal potential of the middle space, relation (19), can be rewritten in the more convenient form

(23) $$U_{int}\left(r\right)=-\chi_{e}\sum_{l=0}^{\infty }\sum_{m=-l}^{l}\left(C^{\left(l,m\right)}A_{ext}^{\left(l,m\right)}N_{l}\left(r^{l}-\frac{a^{2l+1}}{r^{l+1}}\right)+D^{\left(l,m\right)}N_{l+1}\left(-\frac{r^{l}}{b^{2l+1}}+\frac{1}{r^{l+1}}\right)\right)Y_{l}^{m}\left(\theta ,\varphi \right)$$

The respective internal electric field of the middle space is obtained through relation E(r)=−∇U(r). From relation (23) we obtain the following relation:

(24) $$E_{int}\left(r\right)=\left\{\chi_{e}\sum_{l=0}^{\infty }\sum_{m=-l}^{l}\left(C^{\left(l,m\right)}A_{ext}^{\left(l,m\right)}N_{l}\left(lr^{l-1}+\left(l+1\right)\frac{a^{2l+1}}{r^{l+2}}\right)-D^{\left(l,m\right)}N_{l+1}\left(l\frac{r^{l-1}}{b^{2l+1}}+\left(l+1\right)\frac{1}{r^{l+2}}\right)\right)Y_{l}^{m}\left(\theta ,\varphi \right)\right\}\hat{r}\;+\chi_{e}\sum_{l=0}^{\infty }\sum_{m=-l}^{l}\left\{\left(C^{\left(l,m\right)}A_{ext}^{\left(l,m\right)}N_{l}\left(r^{l-1}-\frac{a^{2l+1}}{r^{l+2}}\right)+D^{\left(l,m\right)}N_{l+1}\left(-\frac{r^{l-1}}{b^{2l+1}}+\frac{1}{r^{l+2}}\right)\right)\left(\nabla_{\theta \varphi }Y_{l}^{m}\left(\theta ,\varphi \right)\right)\right\}$$

where the gradient over the angles, $\nabla_{\theta \varphi }$, is defined through the relation $\nabla =\hat{r}(\partial /\partial r)+(1/r)\nabla_{\theta \varphi }$ with $\nabla_{\theta \varphi }=\hat{\theta }\left(\partial /\partial \theta \right)+\hat{\varphi }(1/sin\theta )(\partial /\partial \varphi )$.

Now we are ready to go through the final step G of Figure 2. The total potential of the middle space is simply given by the sum of relations (4) and (23), $U_{tot}\left(r\right)=U_{ext}\left(r\right)+U_{int}\left(r\right)$. We easily obtain the convenient form.

(25) $$U_{tot}\left(r\right)=-\chi_{e}\sum_{l=0}^{\infty }\sum_{m=-l}^{l}(\left(\left(C^{\left(l,m\right)}N_{l}-\chi_{e}^{-1}\right)A_{ext}^{\left(l,m\right)}-D^{\left(l,m\right)}N_{l+1}b^{-\left(2l+1\right)}\right)r^{l}\;\;-\left(C^{\left(l,m\right)}A_{ext}^{\left(l,m\right)}N_{l}a^{2l+1}-D^{\left(l,m\right)}N_{l+1}\right)r^{-\left(l+1\right)})Y_{l}^{m}\left(\theta ,\varphi \right)$$

The respective total electric field of the middle space is obtained through relation E(r)=−∇U(r) and relation (25), providing the following one:

(26) $$E_{tot}\left(r\right)=\chi_{e}\sum_{l=0}^{\infty }\sum_{m=-l}^{l}\left\{\left(\left(C^{\left(l,m\right)}N_{l}-\chi_{e}^{-1}\right)A_{ext}^{\left(l,m\right)}-D^{\left(l,m\right)}N_{l+1}b^{-\left(2l+1\right)}\right)\left(lr^{l-1}Y_{l}^{m}\left(\theta ,\varphi \right)\hat{r}+r^{l-1}\left(\nabla_{\theta \varphi }Y_{l}^{m}\left(\theta ,\varphi \right)\right)\right)\;+\left(C^{\left(l,m\right)}A_{ext}^{\left(l,m\right)}N_{l}a^{2l+1}-D^{\left(l,m\right)}N_{l+1}\right)\left(\left(l+1\right)r^{-\left(l+2\right)}Y_{l}^{m}\left(\theta ,\varphi \right)\hat{r}-r^{-\left(l+2\right)}\left(\nabla_{\theta \varphi }Y_{l}^{m}\left(\theta ,\varphi \right)\right)\right)\right\}$$

where, again, the gradient over the angles, $\nabla_{\theta \varphi }$, is defined through the relation $\nabla =\hat{r}(\partial /\partial r)+(1/r)\nabla_{\theta \varphi }$ with $\nabla_{\theta \varphi }=\hat{\theta }\left(\partial /\partial \theta \right)+\hat{\varphi }(1/sin\theta )(\partial /\partial \varphi )$.

Once we have obtained the second expression of the total potential of the middle space, relation (25), we equate it with the starting expression, relation (9), asking for a self-consistent solution of the problem. This is a recursive maneuver toward the original linear-based solution. This is why our approach is termed MLRS of the Laplace equation. After some simple algebra we obtain the following expressions for the, until now, unknown linearity coefficients of the middle space

(27) $$C^{\left(l\right)}=\frac{1}{1+N_{l}\chi_{e}\left(1-N_{l+1}\chi_{e,l+1}^{ext}{\left(\frac{a}{b}\right)}^{2l+1}\right)}$$

and

(28) $$D^{\left(l\right)}=\frac{N_{l}\chi_{e,l+1}^{ext}}{1+N_{l}\chi_{e}\left(1-N_{l+1}\chi_{e,l+1}^{ext}{\left(\frac{a}{b}\right)}^{2l+1}\right)}a^{2l+1}A_{ext}^{\left(l,m\right)}$$

From relation (27) we see that the coefficients, $C^{\left(l\right)}$, are dimensionless. On the contrary, from relation (28) we see that the coefficients, $D^{\left(l\right)}$, depend on the inner radius of the spherical hollow shell, $a^{2l+1}$, and on the coefficients of the external potential, $A_{ext}^{\left(l,m\right)}$. Based on physical grounds, we can redefine the coefficients, $D^{\left(l\right)}$, to become dimensionless, as well. This can be done by rejecting the term $a^{2l+1}A_{ext}^{\left(l,m\right)}$ from relation (28), while we directly incorporate it in relations (31) and (32), of the total, $U_{tot}\left(r\right)$, and internal, $U_{int}\left(r\right)$, potential, respectively (see below). Thus, for the coefficients $D^{\left(l\right)}$ we have the dimensionless expression given by the relation

(29) $$D^{\left(l\right)}=\frac{N_{l}\chi_{e,l+1}^{ext}}{1+N_{l}\chi_{e}\left(1-N_{l+1}\chi_{e,l+1}^{ext}{\left(\frac{a}{b}\right)}^{2l+1}\right)}$$

In these relations we have dropped the upper index of the order, m, since the linearity coefficients are degenerate over the 2l+1 available values of the order m, −l≤m≤l. Also, we have introduced the following definition for the extrinsic electric susceptibility of degree l+1, $\chi_{e,l+1}^{ext}$, that is valid for any form of the external electric potential, $U_{ext}\left(r\right)$, by the relation

(30) $$\chi_{e,l+1}^{ext}=\frac{\chi_{e}^{int}}{1+N_{l+1}\chi_{e}^{int}}$$

where the depolarization factor, $N_{l+1}$, of degree, l+1, has already been defined through relation (22). *The above relation (30) in connection to relation (21) clarifies that the two distinct depolarization factors*, $N_{l}$, *and*, $N_{l+1}$, *are accompanied by the two relevant extrinsic susceptibilities*, $\chi_{e,l}^{ext}$, *and*, $\chi_{e,l+1}^{ext}$*. Importantly*, *all* $N_{l}$, $N_{l+1}$, $\chi_{e,l}^{ext}$ *and* $\chi_{e,l+1}^{ext}$ *are degenerate on the order*, m, *of the mode of the external potential*, $U_{ext}^{\left(l,m\right)}\left(r\right)$.

To summarize, following the above definitions of the linearity coefficients, relations (27) and (29), aided by relations (20)–(22) and (30), we conclude that the total electric potential of the middle space, $U_{tot}\left(r\right)$, is given by relation (9), reproduced here for convenience

(31) $$U_{tot}\left(r\right)=\sum_{l=0}^{\infty }\sum_{m=-l}^{l}C^{\left(l\right)}r^{l}A_{ext}^{\left(l,m\right)}Y_{l}^{m}(\theta ,\varphi )+\sum_{l=0}^{\infty }\sum_{m=-l}^{l}D^{\left(l\right)}a^{2l+1}r^{-\left(l+1\right)}A_{ext}^{\left(l,m\right)}Y_{l}^{m}(\theta ,\varphi )$$

Also, the internal electric potential of the middle space, $U_{int}\left(r\right)$, follows the analogous relation

(32) $$U_{int}\left(r\right)=\sum_{l=0}^{\infty }\sum_{m=-l}^{l}\left(C^{\left(l\right)}-1\right)r^{l}A_{ext}^{\left(l,m\right)}Y_{l}^{m}(\theta ,\varphi )+\sum_{l=0}^{\infty }\sum_{m=-l}^{l}D^{\left(l\right)}a^{2l+1}r^{-\left(l+1\right)}A_{ext}^{\left(l,m\right)}Y_{l}^{m}(\theta ,\varphi )$$

Interestingly, by a close inspection of relations (27) and (29) we see that $D^{\left(l\right)}=C^{\left(l\right)}N_{l}\chi_{e,l+1}^{ext}$. Thus, it is convenient to express, $U_{tot}\left(r\right)$, and, $U_{int}\left(r\right)$, solely through the coefficients, $C^{\left(l\right)}$. Accordingly, relations (31) and (32) can be rewritten in the more compact and informative form

(33) $$U_{tot}\left(r\right)=\sum_{l=0}^{\infty }C^{\left(l\right)}\left(r^{l}+N_{l}\chi_{e,l+1}^{ext}a^{2l+1}r^{-\left(l+1\right)}\right)\sum_{m=-l}^{l}A_{ext}^{\left(l,m\right)}Y_{l}^{m}(\theta ,\varphi )$$

and

(34) $$U_{int}\left(r\right)=\sum_{l=0}^{\infty }\left(C^{\left(l\right)}\left(r^{l}+N_{l}\chi_{e,l+1}^{ext}a^{2l+1}r^{-\left(l+1\right)}\right)-r^{l}\right)\sum_{m=-l}^{l}A_{ext}^{\left(l,m\right)}Y_{l}^{m}(\theta ,\varphi )$$

The respective, total and internal electric fields of the middle space are simply given by the gradient, E(r)=−∇U(r), of the above relations (33) and (34).

Also, we see that the expansion coefficients, $A_{ext}^{\left(l,m\right)}$, of the external potential, $U_{ext}(r)$ (given by relation (6)) appear in the expressions of the total and internal electric potentials/fields. Thus, relation (6) that actually determines the existing modes (l,m) of the external potential, $U_{ext}\left(r\right)$, i.e., the non-zero values of (l,m), will also determine the existing modes of the total and internal potential in the middle space of the spherical hollow shell.

Finally, once the total and internal potentials are known, all other important physical entities of the middle space are also known. The electric polarization, P(r), is given by relation (11), while the two surface densities of bound charges, $\sigma_{b}\left(r\right){|}_{r=a}$ and $\sigma_{b}\left(r\right){|}_{r=b}$, that reside at the inner, r=a, and outer, r=b, surface of the dielectric spherical hollow shell are given by relations (12) and (13), respectively. These physical entities can be utilized in applications. For instance, from the polarization of the middle space we can calculate the electric dipole moment, p, of the spherical hollow shell through the polarization, P(r), by using $p=\int_{V}P(r)dV$. Then, both the force, F, and the torque, T, acting on the spherical hollow shell can be calculated through the standard relations, $F=(p\cdot \nabla )E_{ext}(r)$ and $T=p\times E_{ext}(r)$, respectively.

### 3.2. Dielectric Spherical Hollow Shell: Inside (0≤r≤a) and Outside (b≤r) Spaces

In this subsection we present the results for the inside (0≤r≤a) and outside (b≤r) spaces of the dielectric spherical hollow shell. Any calculations on the internal field in these spaces should rely on the secondary sources of bound charges, $\sigma_{b}\left(r\right){|}_{r=a}$ and $\sigma_{b}\left(r\right){|}_{r=b}$, that reside at the inner, r=a, and outer, r=b, surfaces. These are known, relations (12) and (13), respectively, while aided by relations (27) and (28). These calculations can directly be based on the generalized law of Coulomb, as done above for the middle space. Equivalently, the superposition principle, $U_{tot}\left(r\right)=U_{ext}\left(r\right)+U_{int}\left(r\right)$ can be employed.

#### 3.2.1. Inside Space (0≤r≤a) of the Dielectric Spherical Hollow Shell

For the inside space, 0≤r≤a, the following relations are obtained

(35) $$U_{int}\left(r\right)=\sum_{l=0}^{\infty }\left(C^{\left(l\right)}-1\right)\sum_{m=-l}^{l}A_{ext}^{\left(l,m\right)}r^{l}Y_{l}^{m}(\theta ,\varphi )$$

and

(36) $$U_{tot}\left(r\right)=\sum_{l=0}^{\infty }C^{\left(l\right)}\sum_{m=-l}^{l}A_{ext}^{\left(l,m\right)}r^{l}Y_{l}^{m}(\theta ,\varphi )$$

for the internal and total electric potential, respectively. In these relations the linearity coefficients, $C^{\left(l\right)}$, of the inside space are given by the relation

(37) $$C^{\left(l\right)}=\frac{\frac{\chi_{e}+1}{\chi_{e}}\chi_{e,l+1}^{ext}}{1+N_{l}\chi_{e}\left(1-N_{l+1}\chi_{e,l+1}^{ext}{\left(\frac{a}{b}\right)}^{2l+1}\right)}$$

Clearly, relations (35) and (36) are based on the general term of the external potential, $U_{ext}^{\left(l,m\right)}(r)=A_{ext}^{\left(l,m\right)}r^{l}Y_{l}^{m}(\theta ,\varphi )$ (see relations (4)–(6)). Thus, the linearity coefficients, $C^{\left(l\right)}$, can be termed as transmission coefficients of the external potential to the inside space of the hollow shell or shielding coefficients of the inside space of the hollow shell from the external potential, depending on the preferable point of view. Indeed, the linearity coefficients, $C^{\left(l\right)}$, of relation (37), aided by relations (20), (22) and (30), give direct quantitative information on the percentage of screening that each mode of the external potential, $U_{ext}^{\left(l,m\right)}\left(r\right)=A_{ext}^{\left(l,m\right)}r^{l}Y_{l}^{m}(\theta ,\varphi )$, experiences by the respective mode of the internal potential, $U_{int}^{\left(l,m\right)}\left(r\right)=\left(C^{\left(l\right)}-1\right)A_{ext}^{\left(l,m\right)}r^{l}Y_{l}^{m}(\theta ,\varphi )$. This information is important in shielding applications of a space of interest from an external electric potential/field. In Section 6, “Importance of Our Work and Utilization of Our Analytical Results in Applications”, we present relevant simulations on the linearity/transmission/shielding coefficients, $C^{\left(l\right)}$, of relation (37).

Obviously, the relevant internal and total electric fields of the inside space can easily be obtained by using relations (35) and (36), respectively, through the standard relation, E(r)=−∇U(r).

#### 3.2.2. Outside Space (b≤r) of the Dielectric Spherical Hollow Shell

For the outside space, b≤r, the following relations are obtained

(38) $$U_{int}\left(r\right)=\sum_{l=0}^{\infty }D^{\left(l\right)}\sum_{m=-l}^{l}A_{ext}^{\left(l,m\right)}b^{2l+1}r^{-\left(l+1\right)}Y_{l}^{m}(\theta ,\varphi )$$

and

(39) $$U_{tot}\left(r\right)=\sum_{l=0}^{\infty }\sum_{m=-l}^{l}A_{ext}^{\left(l,m\right)}\left(r^{l}+D^{\left(l\right)}b^{2l+1}r^{-\left(l+1\right)}\right)Y_{l}^{m}(\theta ,\varphi )$$

for the internal and total electric potential, respectively. In these relations the linearity coefficients, $D^{\left(l\right)}$, of the outside space are given by the relation

(40) $$D^{\left(l\right)}=-\frac{N_{l}\chi_{e}\left(1-{\left(\frac{a}{b}\right)}^{2l+1}\right)}{1+N_{l}\chi_{e}\left(1-N_{l+1}\chi_{e,l+1}^{ext}{\left(\frac{a}{b}\right)}^{2l+1}\right)}$$

Clearly, relations (38) and (39) show how much each mode of the external potential, $U_{ext}^{\left(l,m\right)}(r)=A_{ext}^{\left(l,m\right)}r^{l}Y_{l}^{m}(\theta ,\varphi )$, is modified in the outside space of the hollow shell by the respective mode of the internal potential, $U_{int}^{\left(l,m\right)}(r)=D^{\left(l\right)}A_{ext}^{\left(l,m\right)}b^{2l+1}r^{-\left(l+1\right)}Y_{l}^{m}(\theta ,\varphi )$, that the hollow shell produces in response to $U_{ext}^{\left(l,m\right)}(r)$. Thus, the linearity coefficients, $D^{\left(l\right)}$, can be termed as distortion coefficients of the external potential. Indeed, the linearity coefficients, $D^{\left(l\right)}$, of relation (40), aided by relations (20), (22) and (30), give direct quantitative information on the percentage of distortion that each mode of the external potential, $U_{ext}^{\left(l,m\right)}(r)$, experiences at the outside space due to the respective mode of the internal potential, $U_{int}^{\left(l,m\right)}(r)$. This information is important in invisibility applications where an object of interest should not be detected by an external electric potential/field. In Section 6, “Importance of Our Work and Utilization of Our Analytical Results in Applications”, we present relevant simulations on the linearity/distortion coefficients, $D^{\left(l\right)}$, of relation (40). Obviously, the relevant internal and total electric fields of the outside space can easily be obtained by using relations (38) and (39), respectively, through the standard relation, E(r)=−∇U(r).

## 4. Magnetic Spherical Hollow Shell: Inside (0≤r≤a), Middle (a≤r≤b) and Outside (b≤r) Spaces

In this subsection we discuss the spherical hollow shell consisting of linear, homogeneous and isotropic magnetic material of constant susceptibility, $\chi_{m}$. We follow the same procedure, outlined in Figure 2, as utilized above for the dielectric material. Since we follow the exact same MLRS, details are omitted so that we directly present the results for the inside (0≤r≤a), middle (a≤r≤b) and outside (b≤r) spaces of the magnetic spherical hollow shell.

### 4.1. Inside Space (0≤r≤a) of the Magnetic Spherical Hollow Shell

The internal and total magnetic pseudopotentials of the inside space, 0≤r≤a, are given by the relations

(41) $$U_{int}\left(r\right)=\sum_{l=0}^{\infty }\left(C^{\left(l\right)}-1\right)\sum_{m=-l}^{l}A_{ext}^{\left(l,m\right)}r^{l}Y_{l}^{m}(\theta ,\varphi )$$

and

(42) $$U_{tot}\left(r\right)=\sum_{l=0}^{\infty }C^{\left(l\right)}\sum_{m=-l}^{l}A_{ext}^{\left(l,m\right)}r^{l}Y_{l}^{m}(\theta ,\varphi )$$

In these relations the linearity coefficients, $C^{\left(l\right)}$, of the inside space are given by the relation

(43) $$C^{\left(l\right)}=\frac{\frac{\chi_{m}+1}{\chi_{m}}\chi_{m,l+1}^{ext}}{1+N_{l}\chi_{m}\left(1-N_{l+1}\chi_{m,l+1}^{ext}{\left(\frac{a}{b}\right)}^{2l+1}\right)}$$

The physical meaning of the linearity coefficients, $C^{\left(l\right)}$, of the inside space for the magnetic case studied in this subsection, is the same of the respective ones discussed for the dielectric case in the above Section 3.2 “Dielectric Spherical Hollow Shell-Inside (0≤r≤a) and Outside (b≤r) Spaces”. Thus, the linearity coefficients, $C^{\left(l\right)}$, of relation (43), aided by relations (20), (22) and (30), give direct quantitative information on the percentage of screening that each mode of the external pseudopotential, $U_{ext}^{\left(l,m\right)}\left(r\right)=A_{ext}^{\left(l,m\right)}r^{l}Y_{l}^{m}(\theta ,\varphi )$, experiences by the respective mode of the internal pseudopotential, $U_{int}^{\left(l,m\right)}\left(r\right)=\left(C^{\left(l\right)}-1\right)A_{ext}^{\left(l,m\right)}r^{l}Y_{l}^{m}(\theta ,\varphi )$. This information is important in shielding applications of a space of interest from an external magnetic pseudopotential/field.

Obviously, the relevant internal and total magnetic fields of the inside space can easily be obtained by using relations (41) and (42), respectively, through the standard relation, H(r)=−∇U(r).

### 4.2. Middle Space (a≤r≤b) of the Magnetic Spherical Hollow Shell

The internal and total magnetic pseudopotentials of the middle space, a≤r≤b, are given by the relations

(44) $$U_{tot}\left(r\right)=\sum_{l=0}^{\infty }C^{\left(l\right)}\sum_{m=-l}^{l}A_{ext}^{\left(l,m\right)}\left(r^{l}+N_{l}\chi_{m,l+1}^{ext}a^{2l+1}r^{-\left(l+1\right)}\right)Y_{l}^{m}(\theta ,\varphi )$$

and

(45) $$U_{int}\left(r\right)=\sum_{l=0}^{\infty }C^{\left(l\right)}\sum_{m=-l}^{l}A_{ext}^{\left(l,m\right)}\left(\left(r^{l}+N_{l}\chi_{m,l+1}^{ext}a^{2l+1}r^{-\left(l+1\right)}\right)-r^{l}\right)Y_{l}^{m}(\theta ,\varphi )$$

where

(46) $$C^{\left(l\right)}=\frac{1}{1+N_{l}\chi_{m}\left(1-N_{l+1}\chi_{m,l+1}^{ext}{\left(\frac{a}{b}\right)}^{2l+1}\right)}$$

The relevant total and internal magnetic fields of the middle space are simply given through the relevant pseudopotential by the gradient, H(r)=−∇U(r), of the above relations (44) and (45), respectively. The magnetic polarization, M(r), and the two surface densities of bound magnetic pseudocharges, $\sigma_{b}\left(r\right){|}_{r=a}$ and $\sigma_{b}\left(r\right){|}_{r=b}$, that reside at the inner, r=a, and outer, r=b, surface of the magnetic spherical hollow shell are easily obtained through standard expressions. These analytical results can immediately be utilized in applications.

### 4.3. Outside Space (b≤r) of the Magnetic Spherical Hollow Shell

The internal and total magnetic pseudopotentials of the outside space, b≤r, are given by the relations

(47) $$U_{int}\left(r\right)=\sum_{l=0}^{\infty }D^{\left(l\right)}\sum_{m=-l}^{l}A_{ext}^{\left(l,m\right)}b^{2l+1}r^{-\left(l+1\right)}Y_{l}^{m}(\theta ,\varphi )$$

and

(48) $$U_{tot}\left(r\right)=\sum_{l=0}^{\infty }\sum_{m=-l}^{l}A_{ext}^{\left(l,m\right)}\left(r^{l}+D^{\left(l\right)}b^{2l+1}r^{-\left(l+1\right)}\right)Y_{l}^{m}(\theta ,\varphi )$$

In these relations the linearity coefficients, $D^{\left(l\right)}$, of the outside space are given by the relation

(49) $$D^{\left(l\right)}=-\frac{N_{l}\chi_{m}\left(1-{\left(\frac{a}{b}\right)}^{2l+1}\right)}{1+N_{l}\chi_{m}\left(1-N_{l+1}\chi_{m,l+1}^{ext}{\left(\frac{a}{b}\right)}^{2l+1}\right)}$$

The physical meaning of the linearity coefficients, $C^{\left(l\right)}$, of the outside space for the magnetic case studied in this subsection, is the same of the respective ones discussed for the dielectric case in the above Section 3.2 “Dielectric Spherical Hollow Shell-Inside (0≤r≤a) and Outside (b≤r) Spaces”. Thus, the linearity coefficients, $D^{\left(l\right)}$, of relation (49), aided by relations (20), (22) and (30), give direct quantitative information on the percentage of distortion that each mode of the external pseudopotential, $U_{ext}^{\left(l,m\right)}\left(r\right)=A_{ext}^{\left(l,m\right)}r^{l}Y_{l}^{m}(\theta ,\varphi )$, experiences by the respective mode of the internal pseudopotential, $U_{int}^{\left(l,m\right)}\left(r\right)=D^{\left(l\right)}A_{ext}^{\left(l,m\right)}b^{2l+1}r^{-\left(l+1\right)}Y_{l}^{m}(\theta ,\varphi )$. This information is important in invisibility applications where an object of interest should not be detected by an external magnetic pseudopotential/field.

Obviously, the relevant internal and total magnetic fields of the outside space can easily be obtained by using relations (47) and (48), respectively, through the standard relation, H(r)=−∇U(r).

## 5. The Case of a Homogeneous Dielectric Solid Sphere

Here we investigate how the above relations transform for the case where the spherical hollow shell becomes solid, that is when a=0. These results can be directly compared to the ones obtained in [44,45] for solid spheres consisting of linear, homogeneous and isotropic dielectric and magnetic material.

### 5.1. Comparison of the Dielectric Spherical Building Units

When we consider a=0 so that the dielectric spherical hollow shell becomes a solid sphere, the results of the middle space studied here should be compared to the ones of the inside space studied in [44,45]. Then, relations (33) and (34) referring to the total, $U_{tot}\left(r\right)$, and internal, $U_{int}\left(r\right)$, potential, respectively, get identical to relations (45) and (44) obtained in [45]. In respect to the outside space, relations (38) and (39) referring to the internal, $U_{int}\left(r\right)$, and total, $U_{tot}\left(r\right)$, potential, respectively, get identical to relations (51) and (52) obtained in [45].

### 5.2. Comparison of the Magnetic Spherical Building Units

In the same context, when we consider a=0 so that the magnetic spherical hollow shell becomes a solid sphere, the results of the middle space studied here should be contrasted to the ones of the inside space studied in [44,45]. Then, relations (44) and (45) referring to the total, $U_{tot}\left(r\right)$, and internal, $U_{int}\left(r\right)$, pseudopotential, respectively, get identical to relations (65) and (64) obtained in [45]. In respect to the outside space, relations (47) and (48) referring to the internal, $U_{int}\left(r\right)$, and total, $U_{tot}\left(r\right)$, pseudopotential, respectively, get identical to relations (71) and (72) obtained in [45].

## 6. Importance of Our Work and Utilization of Our Analytical Results in Applications

Our analytical results constitute an important contribution to the literature since they explore in detail both dielectric and magnetic spherical hollow shells on a common mathematical basis by means of the flexible and reliable MLRS of the Laplace equation. The obtained results are of immediate utilization in all relevant applications, for the static and quasistatic limit (dc and ac of low frequency, respectively), where dielectric and magnetic spherical hollow shells are employed. Some applications referring to electro/magneto-phoresis, scattering, invisibility cloaks/shielding, etc. were discussed in Section 1, ‘Introduction’. In such applications the dielectric and magnetic spherical hollow shells can be represented by their dipole moment, p/m, that can be calculated directly from the electric/magnetic polarization, P(r)/M(r), already known for all cases. Once p/m are known, both the force, F, and torque, T, acting in these building units can be calculated straightforwardly. These quantities, F and T, are cornerstone for the design and realization of applications. Below we discuss some salient features and technical advantages of our theoretical results.

First, we refer to the depolarization effect, quantified by the so-called depolarization factor [19,23,26,27,28,29,30,31,32]. We note that in the literature this topic is typically addressed for solid building units, e.g., spheres and cylinders (for spherical hollow shells, see below), almost exclusively for the case where the external field is homogeneous. Also, in recent publications of ours we have discussed this topic on a common basis for both dielectric and magnetic solid spheres [44,45] and cylinders [46]. Referring to solid spheres, in [44,45] we have introduced the depolarization factor, $N_{l}$, and extrinsic susceptibility, $\chi_{e,l}^{ext}$, both ascribed to the respective mode (l,m) of the applied external potential, $U_{ext}\left(r\right)$. In those works [44,45] we proved that both $N_{l}$ and $\chi_{e,l}^{ext}$ are characterized only by the degree, l, of each mode (l,m) since they are degenerate over the different, 2l+1, values of the order, m. In the present work that refers to spherical hollow shells we have also derived $N_{l}$ and $\chi_{e,l}^{ext}$ as relation (20) and (21), respectively. Most importantly, the present work of ours extends previous studies by introducing new physical parameters. Specifically, in addition to $N_{l}$, here we introduce another depolarization factor, $N_{l+1}$, given by relation (22). Also, we have introduced another extrinsic susceptibility, $\chi_{e,l+1}^{ext}$, given by relation (30). Our results, obtained through the MLRS, clearly prove that the depolarization factor, $N_{l}$, and the respective extrinsic susceptibility, $\chi_{e,l}^{ext}$, stem from the outer surface, r=b, while $N_{l+1}$ and $\chi_{e,l+1}^{ext}$ originate from the inner surface, r=a, of the spherical hollow shell. Our results document that all $N_{l}$, $N_{l+1}$, $\chi_{e,l}^{ext}$ and $\chi_{e,l+1}^{ext}$ are degenerate on the order, m, of the mode of the external potential, $U_{ext}^{\left(l,m\right)}\left(r\right)$. Importantly, the sum of these two distinct depolarization factors equals unity, $N_{l}+N_{l+1}=1$, irrespective of the degree, l, of the mode of the external potential, $U_{ext}^{\left(l,m\right)}\left(r\right)$.

Second, we have to clarify that all theoretical relations introduced in this work have a clear physical meaning. To this end we have to clarify that all above relations on the internal and total scalar potentials/pseudopotentials, internal and total vector electric/magnetic fields, electric/magnetic polarization and surface densities of bound charges/pseudocharges always result in a real output, as they should. Indeed, referring to the spherical hollow shells, in all above relations the summation over the, 2l+1, values of the order m, −l≤m≤l, always includes pairs of the following form $\left(A_{ext}^{\left(l,m\right)}Y_{l}^{m}\left(\theta ,\varphi \right)+A_{ext}^{\left(l,-m\right)}Y_{l}^{-m}\left(\theta ,\varphi \right)\right)$. These pairs always result in a real output equal to $2R\left(A_{ext}^{\left(l,m\right)}Y_{l}^{m}\left(\theta ,\varphi \right)\right)$, where R, denotes the real part of the complex, $A_{ext}^{\left(l,m\right)}Y_{l}^{m}\left(\theta ,\varphi \right)$, as was evidenced in detail in Appendix A of [44].

Third, we underline that our analytical results can be adjusted immediately to the specific characteristics of each problem/application. This is done at the very beginning of each case since the external potential, $U_{ext}\left(r\right)$, is directly expanded on the basis of SH. This expansion determines the valid, i.e., non-zero, modes of $U_{ext}\left(r\right)$ so that only these are considered during the application of the MLRS of the Laplace equation. Subsequently, these valid modes enter the analytical relations obtained for all scalar and vector physical entities of interest. Most important, these relations have already metabolized all calculations by proper incorporation of all relevant information, so that they are ready-to-use, without the need to solve each different problem from scratch. This is a noticeable technical advantage for applications.

Fourth, our work extends useful articles published in the literature in some relevant research areas. Referring to shielding applications, our work adds new information on the polarization and the depolarization effect that until now has not been addressed adequately in the literature in hollow shells. Specifically, until now the few publications existing in the literature investigated spherical hollow shells subjected solely to a homogeneous external field [47,48,49]. In [48] J. Prat-Camps et al. investigated the depolarization effect/factor and the magnetic shielding for spherical hollow shells consisting of linear, homogenous and isotropic magnetic material, when subjected to a homogeneous external magnetic field. The authors solved the problem by using the general solution of the Laplace equation in spherical coordinates. The obtained results reproduced consistently standard limiting cases. Very recently, in [49] V.P. Savin and Y.A. Koksharov investigated a more complicated spherical building unit, a core–shell particle that consisted of a single-domain hard ferromagnetic core and a soft magnetic shell made of linear, homogeneous and isotropic material. The core–shell particle was subjected to a homogenous external magnetic field. The authors employed the standard technique of solving the Laplace equation for the magnetic pseudopotential and derived detailed results on the magnetic screening. They also evidenced that the soft magnetic shell acts as an interface that exerts bidirectional screening. The shell screens the core from the external homogeneous magnetic field, while inversely it can also screen the outside space from the magnetic field of the core. Though all above works are useful, they are subjected to the limitation of a homogeneous external field [47,48,49]. This limitation is restored by the present work of ours that investigates these processes for dielectric and magnetic spherical hollow shells subjected to an external potential of *any* spatial form.

In a recent publication [55] A. Lakhtakia and colleagues employed tesseral harmonics to investigate the perturbation of an electrostatic field by a solid sphere consisting of linear and homogeneous, however anisotropic, dielectric material. The authors employed a bijective spatial transformation for the inside space to establish a series representation of the electric potential, while in the outside space they employed the standard form for the solution of both the source and the perturbation potentials. By using the proper boundary conditions on the surface of the solid sphere, they derived a transition matrix that in the outside space relates the expansion coefficients of the perturbation potential to those of the source potential. These analytical calculations were assisted by numerical ones for standard source potentials, e.g., point charge and point dipole. In [56] the authors extended the Farafonov formulation and employed the extended-boundary-condition-method to investigate the perturbation of a source potential by a three-dimensional solid object of irregular shape that consisted of a linear and homogeneous, however anisotropic, dielectric material with positive relative permittivity dyadic. The electric potential at the inside space of the object was represented by a basis obtained through an affine bijective transformation, while at the outside space the electrostatic counterpart of the Ewald–Oseen extinction theorem was recruited. Interestingly, the extended-boundary-condition-method yielded a transition matrix that depended on the geometry and the intrinsic properties of the object, but not on the source potential. In [57] the authors addressed an even more demanding problem. They investigated the perturbation of an electrostatic field by a core-coating solid sphere made of two different linear and homogeneous, however anisotropic, dielectric materials. To this end they utilized the affine spatial transformation which allowed them to use the standard solutions of the Laplace equation. It was found that outside the core-coating solid sphere the expansion coefficients of the perturbation potential related to those of the source potential. The developed method enabled the authors to survey the electric potential inside the core and the coating, as well. Our work investigates similar processes in isotropic dielectric and magnetic spherical hollow shells, and thus it adds complementary information to the above studies [55,56,57].

Finally, beyond the static/quasistatic regime considered here, the broader idea of engineering structured media to control and localize electromagnetic fields also underpins modern topological photonics, including proposals for robust frequency-dependent trapping (“topological rainbow trapping”) [58].

### 6.1. Simulations on Dielectric and Magnetic Spherical Hollow Shells

Until now we have discussed the connection of our work with many relevant applications and works in the literature. To document the usefulness of our analytical results, in the rest of our work we show detailed simulations for both the dielectric and magnetic spherical hollow shell, referring to the inside, middle and outside spaces. These simulations mainly focus on the scalar electric potential since this is the parent function of all other scalar and vector physical entities of interest. However, for the dielectric case some representative simulations of the internal electric field are presented for the middle space where the depolarization effect takes place. In these simulations the magnitude of the external electric field is considered to be dimensionless and normalized, $E_{0}=1$. Detailed simulations and comparison to experimental data drawn from the literature are presented for the magnetic spherical hollow shell, as well. In all simulations shown below we use arbitrary units for the radii, a and b, of the spherical hollow shell (except for cases where clarifications are given).

#### 6.1.1. Inside Space of Dielectric Spherical Hollow Shell

**Figure 3:**
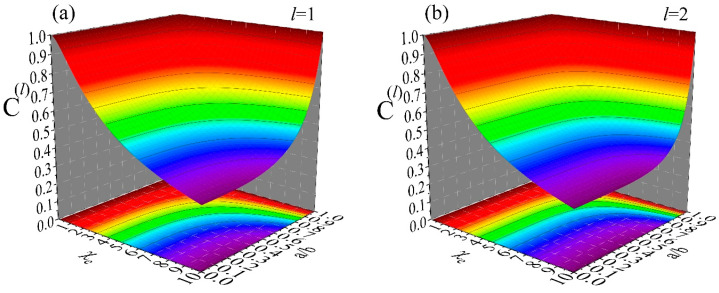

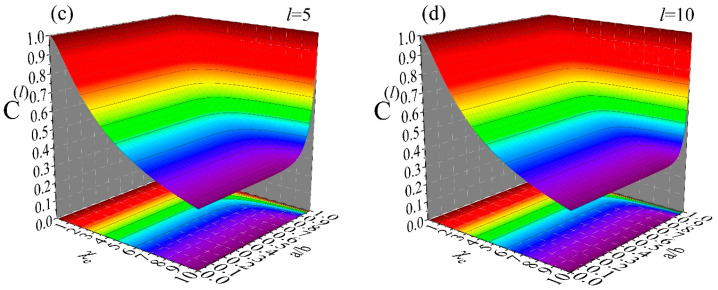
Simulations of the transmission/shielding coefficients, $C^{\left(l\right)}$, relation (37), for a dielectric spherical hollow shell of susceptibility, $\chi_{e}$, inner radius a and outer radius b. The simulations of all panels (**a**–**d**) cover the ranges $0\leq \chi_{e}\leq 10$ and 0≤a/b≤1 and refer to different modes, $U_{ext}^{\left(l,m\right)}(r)$, of the external potential, $U_{ext}(r)$, with degree (**a**) l=1, (**b**) l=2, (**c**) l=5 and (**d**) l=10.

Figure 3 shows simulations for the inside space (0≤r≤a) of a dielectric spherical hollow shell of susceptibility, $\chi_{e}$, inner radius a and outer radius b. These data show how the different modes, $U_{ext}^{\left(l,m\right)}(r)$, of an external potential, $U_{ext}(r)$, reach the inside space of the hollow shell. This is represented by the transmission/shielding coefficients, $C^{\left(l\right)}$, given by relation (37), since this is the coefficient that determines each mode, $U_{tot}^{\left(l,m\right)}(r)$, of the total potential, $U_{tot}(r)$, by multiplying the respective mode, $U_{ext}^{\left(l,m\right)}(r)$, of the external potential, $U_{ext}(r)$ (see relation (36)). Panels (a), (b), (c) and (d) refer to a mode (l,m) of degree l=1, 2, 5 and 10, respectively. All simulations cover the same ranges $0\leq \chi_{e}\leq 10$ and 0≤a/b≤1. These data clearly demonstrate that modes (l,m) of high degree, l, are excluded effectively (low transmission/high shielding) even by thin shells. For instance, to succeed transmission coefficients below a threshold value, e.g., $C^{\left(l\right)}\leq 0.38$, for a material of susceptibility $\chi_{e}=10$, we need a shell with a/b~0.58 when l=1 (panel (a)), a/b~0.77 when l=2 (panel (b)), a/b~0.89 when l=5 (panel (c)) and a/b~0.93 when l=10 (panel (d)). This information can be very important in lightweight applications where the desired performance should be provided by structures of minimum weight and low cost.

#### 6.1.2. Middle Space of Dielectric Spherical Hollow Shell

**Figure 4:**
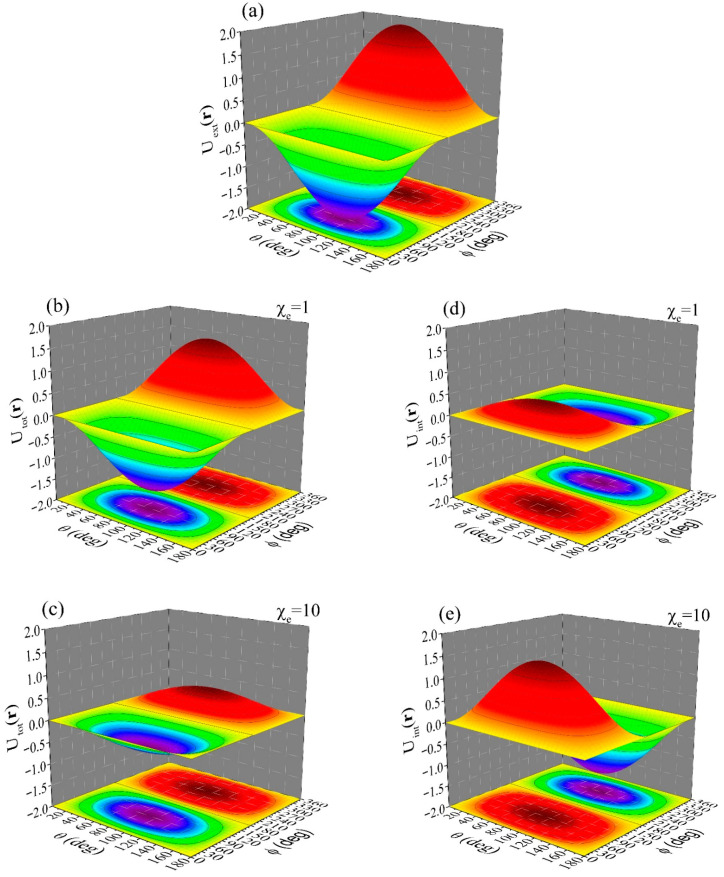
Simulations of the external potential, $U_{ext}\left(r\right)$, total potential, $U_{tot}\left(r\right)$, and internal potential, $U_{int}\left(r\right)$, upon variation in the polar (θ) and azimuthal (φ) angles for a dielectric spherical hollow shell of inner radius a=1 and outer radius b=5, for different values of the susceptibility, $\chi_{e}$. (**a**) Simulation of the applied $U_{ext}\left(r\right)$, relation (50). (**b**,**c**) Simulations of $U_{tot}\left(r\right)$, relation (52), when (**b**) $\chi_{e}=1$ and (**c**) $\chi_{e}=10$. (**d**,**e**) Simulations of $U_{int}\left(r\right)$, relation (51), when (**d**) $\chi_{e}=1$ and (**e**) $\chi_{e}=10$. All simulations refer to the front of radial distance, r, with a=1≤r=2≤b=5, while $E_{0}=1$.

Figure 4 shows simulations for the middle space (a≤r≤b) of a dielectric spherical hollow shell of inner radius, a=1, and outer radius, b=5, constructed by materials of different value of susceptibility, $\chi_{e}$. The building unit is subjected to an external potential given by the relation

(50) $$U_{ext}\left(r\right)=-E_{0}rsin\theta sin\varphi$$

We stress that this case is not exotic. Actually, it is a quite typical one referring to an electric field that is homogeneous, directed along the y axis, $E_{ext}=E_{0}\hat{y}$. We prefer to discuss this typical case since our results can be easily compared to the ones obtained with standard methods. Actually, the effectiveness of our MLRS becomes obvious for more complicated external potentials, $U_{ext}\left(r\right)$. In the case discussed here, by using relations (4)–(6) we can easily verify that $U_{ext}\left(r\right)$ consists of two valid modes (l,m), namely (1,−1) and (1,1). Thus, the respective expansion coefficients, $A_{ext}^{\left(l,m\right)}$, in relation (6), are $A_{ext}^{\left(1,-1\right)}=A_{ext}^{\left(1,1\right)}=-iE_{0}\sqrt{2\pi /3}$. Once the desired $A_{ext}^{\left(l,m\right)}$ are known we can employ relations (34) and (27) (aided by relations (20), (22) and (30)) and directly calculate the internal potential, $U_{int}\left(r\right)$. For the specific case discussed here we have

(51) $$U_{int}\left(r\right)=-\left(\left(C^{\left(1\right)}-1\right)r+C^{\left(1\right)}N_{l}\frac{\chi_{e}}{1+N_{l+1}\chi_{e}}\frac{a^{3}}{r^{2}}\right)E_{0}sin\theta sin\varphi$$

where $N_{l}=1/3$ and $N_{l+1}=2/3$, since l=1. The total potential, $U_{tot}\left(r\right)$, relation (33), is simply obtained by adding $U_{ext}\left(r\right)$ to $U_{int}\left(r\right)$, so that we get

(52) $$U_{tot}\left(r\right)=-C^{\left(1\right)}\left(r+N_{l}\frac{\chi_{e}}{1+N_{l+1}\chi_{e}}\frac{a^{3}}{r^{2}}\right)E_{0}sin\theta sin\varphi$$

In the above relations, the coefficient, $C^{\left(1\right)}$, is given by the following relation

(53) $$C^{\left(1\right)}=\frac{1}{1+N_{l}\chi_{e}\left(1-N_{l+1}\frac{\chi_{e}}{1+N_{l+1}\chi_{e}}{\left(\frac{a}{b}\right)}^{3}\right)}$$

where $N_{l}=1/3$ and $N_{l+1}=2/3$, since l=1.

Panel (a) shows the applied $U_{ext}\left(r\right)$, relation (50). Panels (b) and (c) show the simulations of $U_{tot}\left(r\right)$, relation (52), for $\chi_{e}=1$ and $\chi_{e}=10$, respectively. Panels (d) and (e) show the relevant simulations of $U_{int}\left(r\right)$, relation (51), for $\chi_{e}=1$ and $\chi_{e}=10$, respectively. These simulations refer to the front having a radial distance, r, with a=1≤r=2≤b=5, while we have set $E_{0}=1$. Similar plots can be constructed at different fronts of constant radial distance, r, so that the spatial distribution of $U_{int}\left(r\right)$ and $U_{tot}\left(r\right)$ can be surveyed in the entire middle space (a≤r≤b) of the spherical hollow shell. From these data we see that $U_{int}\left(r\right)$ is opposite to both $U_{tot}\left(r\right)$ and $U_{ext}\left(r\right)$, as expected. Obviously, the respective internal electric field, $E_{int}\left(r\right)$, is opposite to both $E_{tot}\left(r\right)$ and $E_{ext}\left(r\right)$, as well. From these data we see that when $U_{int}\left(r\right)$, panels (d) and (e), adds to $U_{ext}\left(r\right)$, panel (a), the total potential, $U_{tot}\left(r\right)$, is obtained, panels (b) and (c), respectively. Also, for a relatively high value of susceptibility, i.e., $\chi_{e}=10$, $U_{ext}\left(r\right)$ is strongly suppressed by $U_{int}\left(r\right)$, so that $U_{tot}\left(r\right)$ gets practically zero (respective panels (a), (e) and (c)). In such cases where $U_{int}\left(r\right)$ practically equals $U_{ext}\left(r\right)$, the depolarization effect should be very intense.

**Figure 5:**
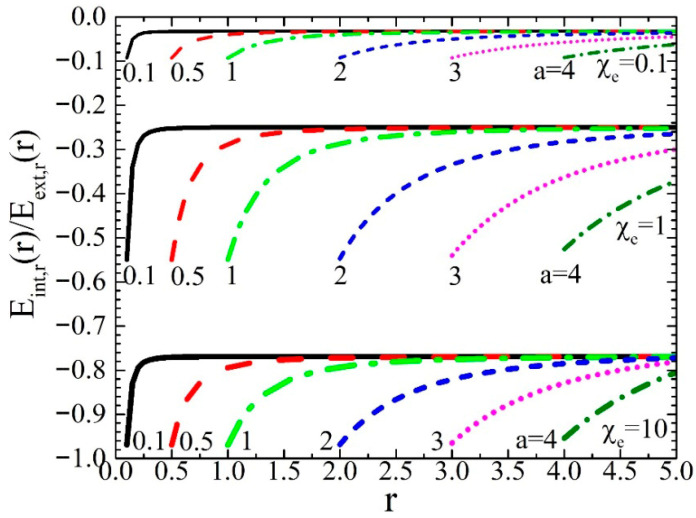
Simulations of the ratio of the radial components of the internal and external electric fields, $E_{int,r}\left(r\right)/E_{ext,r}\left(r\right)$, relation (54), upon variation in the radial distance, r, within a≤r≤b=5. The simulations refer to a dielectric spherical hollow shell of different values of susceptibility, $\chi_{e}=0.1$, upper set of curves, $\chi_{e}=1$, middle set of curves, and $\chi_{e}=10$, lower set of curves. In each set of curves the inner radius, a, takes the values a=0.1, 0.5, 1, 2, 3 and 4 (from left to right).

Since here we discuss the middle space of the dielectric spherical hollow shell we should recall the depolarization effect and make some brief comments. The same external potential, $U_{ext}\left(r\right)$, relation (50), is considered. Probably the most effective way to present our arguments is through simulations of the internal field, $E_{int}\left(r\right)$, in comparison to the external one, $E_{ext}\left(r\right)$ (we recall that $E_{int}\left(r\right)$ is also termed depolarization field, $E_{d}\left(r\right)$, and self-field, $E_{s}\left(r\right)$ [33,34,35,36,37,38]). These can be easily calculated from the relevant potentials, relations (51) and (50), respectively, through the standard relation E(r)=−∇U(r). In the simulations shown below in Figure 5 we focus on the ratio of the radial components of $E_{int}\left(r\right)$ and $E_{ext}\left(r\right)$, given by the relation

(54) $$\frac{E_{int,r}\left(r\right)}{E_{ext,r}\left(r\right)}=\left(C^{\left(1\right)}-1\right)-C^{\left(1\right)}N_{l+1}\frac{\chi_{e}}{1+N_{l+1}\chi_{e}}{\left(\frac{a}{r}\right)}^{3}$$

where $N_{l+1}=2/3$, since l=1. These simulations refer to the case where the outer radius is b=5. Three representative values of the intrinsic susceptibility, $\chi_{e}$, are considered, $\chi_{e}=0.1$, upper set of curves, $\chi_{e}=1$, middle set of curves, and $\chi_{e}=10$, lower set of curves. In each set of curves, we simulate six representative values of the inner radius a=0.1, 0.5, 1, 2, 3 and 4 (from left to right). Obviously, for each value of a the radial distance ranges within a≤r≤b=5.

All these set of data for the different values of the susceptibility, $\chi_{e}$, prove that the radial component of the internal field, $E_{int,r}\left(r\right)$, is always opposite to that of the external one, $E_{ext,r}\left(r\right)$, as it should be (also evidenced for the simulations of Figure 4). In addition, we see that as the inner radius, a, increases towards the outer one, b, the absolute value of the ratio, $\left|E_{int,r}\left(r\right)/E_{ext,r}\left(r\right)\right|$, increases abruptly. This means that as the thickness of the shell decreases, so that the secondary/bound charges that reside at the inner, r=a, and outer, r=b, surfaces of the shell get closer, the radial component of the internal field, $E_{int,r}\left(r\right)$, increases in respect to that of the external one, $E_{ext,r}\left(r\right)$. Obviously, this relates to a stronger depolarization effect in comparison to the case of a solid dielectric sphere. The limiting case refers to an $E_{int,r}\left(r\right)$ that is absolutely directed in the opposite direction of $E_{ext,r}\left(r\right)$, that is $E_{int,r}\left(r\right)=-E_{ext,r}\left(r\right)$. This can be achieved for relatively reasonable values of the susceptibility, $\chi_{e}=10$ (lower set of curves). Thus, under such conditions the depolarization effect should be intense and probably detrimental to specific kinds of applications.

#### 6.1.3. Outside Space of Dielectric Spherical Hollow Shell

**Figure 6:**
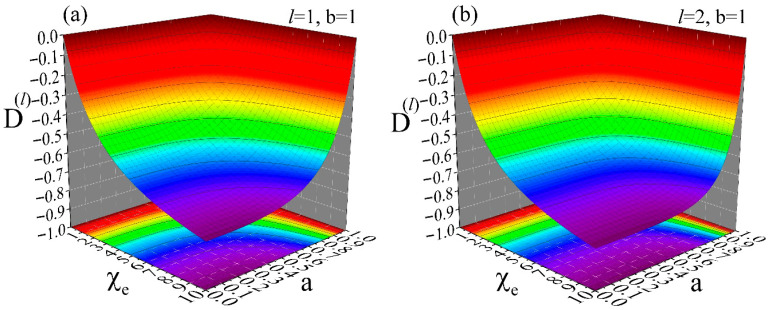

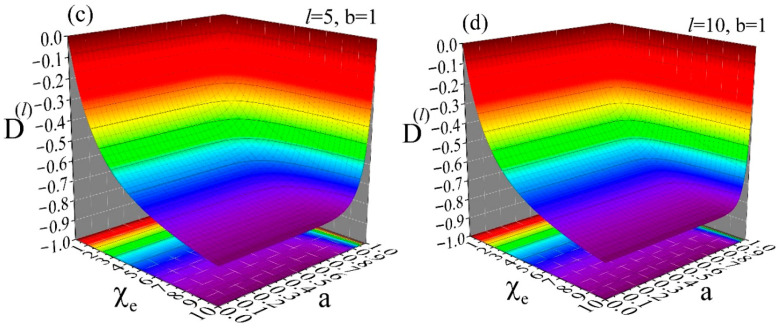
Simulations of the distortion coefficients, $D^{\left(l\right)}$, relation (40), for a dielectric spherical hollow shell of susceptibility, $\chi_{e}$, inner radius a and outer radius b=1. The simulations cover the ranges $0\leq \chi_{e}\leq 10$ and 0≤a≤b and refer to different modes, $U_{ext}^{\left(l,m\right)}(r)$, of the external potential, $U_{ext}(r)$, with degree (**a**) l=1, (**b**) l=2, (**c**) l=5 and (**d**) l=10.

Finally, we turn our interest to the outside space. Figure 6 shows simulations for the outside space (b≤r) of a dielectric spherical hollow shell of susceptibility, $\chi_{e}$, inner radius a and outer radius b=1. These data show how the different modes, $U_{ext}^{\left(l,m\right)}(r)$, of an external potential, $U_{ext}(r)$, are distorted at the outside space of the hollow shell. This is shown by the distortion coefficients, $D^{\left(l\right)}$, given by relation (40), since this is the coefficient that determines each mode of the internal potential, $U_{int}^{\left(l,m\right)}(r)$ (see relation (38)). The distortion of $U_{ext}(r)$ is introduced from $U_{int}(r)$ through the coefficients $D^{\left(l\right)}$, as clearly evidenced by relation (39) that refers to $U_{tot}(r)$; coefficients $D^{\left(l\right)}$ of negligible values will practically result in $U_{tot}\left(r\right)=U_{ext}(r)$. Panels (a), (b), (c) and (d) refer to a mode (l,m) of degree l=1, 2, 5 and 10, respectively. All simulations cover the same ranges $0\leq \chi_{e}\leq 10$ and 0≤a≤b=1. These data clearly demonstrate that modes (l,m) of high degree, l, are distorted strongly even by thin shells. For instance, to succeed distortion coefficients below a threshold value, e.g., $D^{\left(l\right)}\leq -0.22$, for a material of susceptibility $\chi_{e}=10$, we need a shell with a/b~0.960 when l=1 (panel (a)), a/b~0.980 when l=2 (panel (b)), a/b~0.990 when l=5 (panel (c)) and a/b~0.995 when l=10 (panel (d)). As for the case of the transmission/shielding coefficients, $C^{\left(l\right)}$, of the inside space discussed above, this information can be crucial in lightweight applications where the desired performance should be provided by structures of minimum weight and low cost.

The results of the above simulations can provide comparative information for the benefit of applications. For instance, from the analytical expressions of the transmission coefficients, $C^{\left(l\right)}$, relation (37), and the distortion coefficients, $D^{\left(l\right)}$, relation (40), we can realize that there is a conflict between the action of the internal potential, $U_{int}(r)$, at the inside and outside spaces. This is clearly evidenced from a comparison of the simulations presented in Figure 3 and Figure 6. We see that an intense elimination of the external potential, $U_{ext}(r)$, from the inside space will also result in its strong distortion at the outside space. Thus, from simulations such as the ones presented in Figure 3 we can obtain the physical and geometrical characteristics (i.e., susceptibility, $\chi_{e}$, and inner, a, and outer, b, radius of the spherical hollow shell) that will induce adequate suppression of the potential/field to the inside space, while from simulations such as the ones of Figure 6 we can determine the respective parameters that will ensure only minimum distortion of the potential/field at the outside space. A comparison of these simulations can result in the optimum range of parameters that will satisfy these endogenously contradictory requirements.

#### 6.1.4. Application to Resonance Frequencies in Scattering by a Dielectric Spherical Hollow Shell

Here, we assess the application of our analytical relations referring to the dielectric spherical hollow shell with respect to the resonance frequencies obtained in scattering theory. Specifically, here we focus on the relations of the middle space, a≤r≤b, and see how these can be used for the reliable reproduction of the resonance behavior of the two, probably most popular, building units studied in scattering theory: the solid sphere (SS) and the spherical cavity (SC). In a complementary fashion, below we will examine the reliability of our analytical relations at the inside, 0≤r≤a, and outside, b≤r, spaces, eventually validating our theoretical results throughout the entire space of the dielectric spherical hollow shell.

Focusing on the middle space, a≤r≤b, of the spherical hollow shell, it is obvious that the latter should transform to a SS when we set a=0, and to a SC when we apply b→∞. Under these circumstances, the total potential, $U_{tot}\left(r\right)$, of the middle space, relation (33), should reproduce the theoretical results expected for a SS and a SC, in respect to the resonance frequencies observed in scattering.

In the first case we set a=0 to relations (27) and (33) so that the total potential of the SS is given by the following relation

(55) $$U_{tot}^{SS}\left(r\right)=\sum_{l=0}^{\infty }\frac{r^{l}}{1+N_{l}\chi_{e}}\sum_{m=-l}^{l}A_{ext}^{\left(l,m\right)}Y_{l}^{m}(\theta ,\varphi )$$

where the depolarization factor, $N_{l}$, of the outer surface, r=b, is given by relation (20).

In the second case where we apply b→∞ to relations (27) and (33), the total potential of the SC is finally obtained by the following relation

(56) $$U_{tot}^{SC}\left(r\right)=\sum_{l=0}^{\infty }\frac{1}{1+N_{l}\chi_{e}}\frac{(1+N_{l+1}\chi_{e})r^{l}+N_{l}\chi_{e}a^{2l+1}r^{-\left(l+1\right)}}{1+N_{l+1}\chi_{e}}\sum_{m=-l}^{l}A_{ext}^{\left(l,m\right)}Y_{l}^{m}(\theta ,\varphi )$$

where the depolarization factor, $N_{l+1}$, of the inner surface, r=a, is given by relation (22).

The resonance frequencies of these basic scatterers, the SS and the SC, can be estimated even from the electrostatic case studied here [26,59,60,61]. From these relations (55) and (56) we see that the behavior of the total potential is governed by the poles of the denominator, that for the SS are determined by the condition $1+N_{l}\chi_{e}=0$, while for the SC are defined by the additional condition $1+N_{l+1}\chi_{e}=0$. To obtain the resonance frequencies of these basic scatterers by using these conditions, we should employ a model for the description of the relative permittivity, $\varepsilon_{r}\left(\omega \right)$, of the parent dielectric material.

In the general case where both free and bound electrons contribute to the oscillations of plasma, the relative permittivity of the dielectric material is given by the following relation [26]:

(57) $$\varepsilon_{r}\left(\omega \right)=1-\frac{\omega_{p}^{2}}{\omega^{2}+j\gamma \omega }-\frac{\omega_{p}^{2}}{\omega^{2}-\omega_{0}^{2}+j\gamma \omega }$$

where $\omega_{p}=\sqrt{Ne^{2}/m\varepsilon_{0}}$ is the bulk plasma frequency, $\gamma =b_{d}/m$ is the damping frequency and $\omega_{0}=\sqrt{K/m}$ is a characteristic frequency of bound electrons [26]. In this relation the first term is the normalized high-frequency limit, the second term is the Drude of free electrons and the third term is the Lorentz of bound electrons. In the lossless case, γ=0, that is mostly studied in the literature, the first, normalized, and third Lorentz terms of the above relation are given from relation

(58) $$\varepsilon_{r}\left(\omega \right)=1-\frac{\omega_{p}^{2}}{\omega^{2}-\omega_{0}^{2}}$$

Substituting $\chi_{e}\left(\omega \right)=\varepsilon_{r}\left(\omega \right)-1$, to the condition $1+N_{l}\chi_{e}=0$, relation (55), we obtain for the resonance frequencies of the SS the following relation:

(59) $$\omega_{res}^{SS}=\sqrt{\omega_{0}^{2}+\omega_{p}^{2}\frac{l}{2l+1}}=\sqrt{\omega_{0}^{2}+\omega_{p}^{2}N_{l}}$$

Once again, substituting $\chi_{e}\left(\omega \right)=\varepsilon_{r}\left(\omega \right)-1$, to the distinct condition $1+N_{l+1}\chi_{e}=0$, relation (56), we obtain for the resonance frequencies of the SC the following relation:

(60) $$\omega_{res}^{SC}=\sqrt{\omega_{0}^{2}+\omega_{p}^{2}\frac{l+1}{2l+1}}=\sqrt{\omega_{0}^{2}+\omega_{p}^{2}N_{l+1}}$$

To perform a direct comparison with the literature in the above relations we have to replace $\omega_{0}=0$ (Drude model) so that the resonance frequencies of the SS and SC obtain the following form:

(61) $$\omega_{res}^{SS}=\omega_{p}\sqrt{\frac{l}{2l+1}}=\omega_{p}\sqrt{N_{l}}$$

and

(62) $$\omega_{res}^{SC}=\omega_{p}\sqrt{\frac{l+1}{2l+1}}=\omega_{p}\sqrt{N_{l+1}}$$

Indeed, these relations are identical to the ones reported in the literature for a SS and a SC. For instance, see the theoretical relations (9.25) and (9.43) in [59] and the theoretical and experimental results reported in [26,60,61] and references therein.

#### 6.1.5. Application to Characterization of Magnetic Spherical Hollow Shells

Once in all other subsections (Section 6.1.1, Section 6.1.2, Section 6.1.3, Section 6.1.4 and Section 6.1.6) of the present section we refer to dielectric spherical hollow shells, here we test our analytical relations obtained for the magnetic counterpart. Specifically, here we discuss and clarify the applicability of our theoretical results in using various magnetometers [62,63,64,65] for the characterization of magnetic spheres and spherical hollow shells [66,67,68,69,70,71,72,73,74,75,76,77,78,79,80,81,82].

Indeed, our analytical relations can be employed for the proper interpretation of experimental results coming from commercial magnetometers whose principle of operation relies (entirely or partially) on the utilization of pick-up coils [62,63,64,65] (see references in [64,65], as well). For instance, Superconducting Quantum Interference Device (SQUID) magnetometers, at the first stage of their hardware, employ conventional pick-up coils for the detection of magnetic-flux variations (the Josephson junction comes at a later stage) [62]. Also, Vibrating Sample Magnetometers (VSM), whether in longitudinal or in transverse set up, relies for its operation almost exclusively on the detection of magnetic-flux variations by means of conventional pick-up coils, while the specimen performs an oscillation of relatively small amplitude at a set frequency, usually of low value [63]. The same holds for the first- and second-derivative AC Susceptometers (ACS) in which the specimen is stationary during the measurement [64,65]. Specifically, in such magnetometry measurements wherein macroscopic pick-up coils are used for the detection of the magnetic flux originating from the specimen, the latter is almost always modeled, without any reasonable justification, in the ideal form of a point magnetic dipole (PMD) associated with a moment, $m_{d}$ (where the lower index ‘d’ stands for ‘dipole’). Accordingly, the hardware of the magnetometer records the so-called ‘response’ of the specimen (in the form of a spatially/temporarily varying voltage), while its software executes a fitting to derive the moment, $m_{d}$, of the specimen. Obviously, the majority of specimens studied in commercial magnetometers of these types are neither zero-dimensional, i.e., PMDs, nor of symmetric/regular shape. Nevertheless, practically in all cases even the most ineligible specimens are treated as having the ideal characteristics of a PMD of moment, $m_{d}$. By recalling that $m_{d}$ results from integration of M(r) over the volume of the specimen, we easily understand that this is a really crucial point: the model that we will adopt to describe the magnetic polarization, M(r), of the specimen under investigation will determine the accuracy of the obtained experimental result on its moment, $m_{d}$, in comparison to the real spatial dependence of M(r) within its volume. Accordingly, here we argue that the crude approximation of an ideal PMD, that during the experimental stage is ascribed to an even ineligible specimen, can be restored at some degree by using valid analytical relations to model sensibly the magnetic polarization, M(r), of the specimen. This will eventually give a more reliable interpretation to the recorded signal and its connection with the underlying spatial distribution of the magnetic polarization within the volume of the specimen.

For the case of the magnetic spherical hollow shell studied in this subsection, the total potential, $U_{tot}(r)$, of the middle space, a≤r≤b, relation (44), should be employed to find the magnetic field, H(r), through the relation, H(r)=−∇U(r). Then the magnetic polarization is finally obtained from the constitutive relation, $M\left(r\right)=\chi_{m}H\left(r\right)$. Accordingly, the magnetic polarization of the spherical hollow shell is obtained from the following relation

(63) $$M^{SHS}\left(r\right)=-\chi_{m}\sum_{l=0}^{\infty }\sum_{m=-l}^{l}C^{\left(l\right)}A_{ext}^{\left(l,m\right)}\left(\nabla \left(r^{l}Y_{l}^{m}\left(\theta ,\varphi \right)\right)+\left(N_{l}\chi_{m,l+1}^{ext}a^{2l+1}\right)\nabla \left(r^{-\left(l+1\right)}Y_{l}^{m}\left(\theta ,\varphi \right)\right)\right)$$

where the upper index ‘SHS’ stands for ‘spherical hollow shell’, while the coefficients $C^{\left(l\right)}$ of the middle space are given by relation (46). In spherical coordinates the gradient can be expressed by using the notation $\nabla =\nabla_{r}+(1/r)\nabla_{\theta \varphi }$ where the radial, $\nabla_{r}=\hat{r}\partial /\partial r$, and angular, $\nabla_{\theta \varphi }=\hat{\theta }\partial /\partial \theta +(1/sin\theta )\hat{\varphi }\partial /\partial \varphi$, parts are entirely separated from each other. By introducing this notation to the above relation, after some simple algebra, we obtain the following relation for the magnetic polarization:

(64) $$M^{SHS}\left(r\right)=-\chi_{m}\sum_{l=0}^{\infty }\sum_{m=-l}^{l}C^{\left(l\right)}A_{ext}^{\left(l,m\right)}\left[\hat{r}\left(lr^{l-1}-\left(l+1\right)N_{l}\chi_{m,l+1}^{ext}a^{2l+1}r^{-\left(l+2\right)}\right)Y_{l}^{m}\left(\theta ,\varphi \right)\;+\left(r^{l-1}+N_{l}\chi_{m,l+1}^{ext}a^{2l+1}r^{-\left(l+2\right)}\right)\nabla_{\theta \varphi }Y_{l}^{m}\left(\theta ,\varphi \right)\right]$$

The moment, $m_{d}^{SHS}$, of the spherical hollow shell obtained from this generic expression of its magnetic polarization, $M^{SHS}\left(r\right)$, is simply given by integration over its volume from the following relation:

(65) $$m_{d}^{SHS}=\int_{a}^{b}\int_{0}^{2\pi }\int_{0}^{\pi }M^{SHS}\left(r\right)r^{2}sin\theta d\theta d\varphi dr$$

To this end, the radii, a and b, of the spherical hollow shell should be known with high accuracy, e.g., through transmission electron microscopy. In experimental reality where a specimen subjected to characterization includes a collection of spherical hollow shells, we should restrict our analysis to the ideal monodisperse case. Indeed, this hypothesis is really close to experimental reality [66,67,68,69,70,71,72,73,74]. By inserting relation (64) into relation (65) we obtain the following relation for the moment of the spherical hollow shell:

(66) $$m_{d}^{SHS}=-\chi_{m}\sum_{l=0}^{\infty }\sum_{m=-l}^{l}C^{\left(l\right)}A_{ext}^{\left(l,m\right)}\left[\left(\int_{a}^{b}\left(lr^{l+1}-\left(l+1\right)N_{l}\chi_{m,l+1}^{ext}a^{2l+1}r^{-l}\right)dr\int_{0}^{2\pi }\int_{0}^{\pi }\hat{r}Y_{l}^{m}\left(\theta ,\varphi \right)sin\theta d\theta d\varphi \;+\int_{a}^{b}\left(r^{l+1}+N_{l}\chi_{m,l+1}^{ext}a^{2l+1}r^{-l}\right)dr\int_{0}^{2\pi }\int_{0}^{\pi }\nabla_{\theta \varphi }Y_{l}^{m}\left(\theta ,\varphi \right)sin\theta d\theta d\varphi \right)\right]$$

The two integrals over the radial coordinate can be easily obtained. However, for the general case the integrals over the polar and azimuthal angles need some extensive calculations.

Here we focus on the representative case employed in most of the experiments for the characterization of materials, where the external magnetic field is homogeneous, applied along the positive *z*-axis, i.e., $H_{ext}\left(r\right)=H_{0}\hat{z}$ (it can be dc, as done in SQUID magnetometry, or ac of low frequency in our case, as done in ac susceptibility experiments, see [64,65] and references therein). In this particular case the only mode that survives in the expansion of the external potential (see relations (4)–(6)) is the one with (l,m)=(1,0), while the respective coefficient reads $A_{ext}^{\left(1,0\right)}=-\sqrt{4\pi /3}H_{0}$. Using the above relation (66), after some copious calculations, we obtain the following relation for the dipole moment of the magnetic spherical hollow shell:

(67) $$m_{d}^{SHS}=\frac{1}{1+N_{1}\chi_{m}\left(1-N_{1+1}\chi_{m,1+1}^{ext}{\left(\frac{a}{b}\right)}^{3}\right)}\chi_{m}H_{0}\left(\frac{4\pi }{3}\right)(b^{3}-a^{3})\hat{z}$$

where $N_{1}=1/3$ and $N_{1+1}=2/3$ are the depolarization factors of the outer and inner surface of the magnetic spherical hollow shell, given by relations (20) and (22), respectively, for l=1. Also, $\chi_{m,1+1}^{ext}=\chi_{m}/(1+(2/3)\chi_{m})$ is the extrinsic magnetic susceptibility, relation (30), for l=1 (where the lower index ‘e’ is replaced by ‘m’). The above relation (67) can be written in the following compact version:

(68) $$m_{d}^{SHS}=C^{\left(1\right)}\chi_{m}H_{0}V^{SHS}\hat{z}=m_{d}^{SHS}\hat{z}$$

In the above relation (68), $C^{\left(1\right)}$ is the coefficient of the middle space given by relation (46) for l=1, and $V^{SHS}=(4\pi /3)(b^{3}-a^{3})$ is the volume of the spherical hollow shell. Thus, $m_{d}^{SHS}=M_{0}^{cor}V^{SHS}$ can be considered as the magnetic dipole of the spherical hollow shell under the assumption that its magnetic polarization is homogeneous and directed along the *z*-axis, however corrected according to the relation $M_{0}^{cor}=C^{\left(1\right)}\chi_{m}H_{0}\hat{z}$, due to the change in the geometry from a solid sphere to a spherical hollow shell.

Relations (67) and (68) warn us about the careful interpretation of an experimentally recorded dipole moment of a magnetic spherical hollow shell of radii ratio, a/b, and of intrinsic susceptibility, $\chi_{m}$. Specifically, the experimentally determined dipole moment of a magnetic spherical hollow shell, $m_{exp}^{SHS}$, should be equated to the theoretically predicted one, $m_{d}^{SHS}$, via relations (67) and (68), for calculating the intrinsic susceptibility, $\chi_{m}$, of the parent linear, homogeneous and isotropic magnetic material, given that the radii, a and b, are known, e.g., by means of transmission electron microscopy experiments. If instead of the exact dipole moment, $m_{d}^{SHS}$, we employ the crude assumption of a non-corrected, spatially homogeneous magnetic polarization ascribed to a dipole moment, $m_{d0}^{SHS}=\chi_{m}H_{0}V^{SHS}\hat{z}$, i.e., we neglect the prefactor, $C^{\left(1\right)}$, of relations (67) and (68), the obtained value of the intrinsic susceptibility, $\chi_{m}$, will be invalid.

Once we have derived the dipole moment, $m_{d}^{SHS}$, of the magnetic spherical hollow shell, we can now proceed by making a comparison with experimental data on relevant specimens published in the literature. To this end, we recall that in almost all cases the reported dc-magnetometry data refer to the *z*-axis-directed dipole moment of the specimen, i.e., $m_{d}$ (for instance, obtained by means of a SQUID in the longitudinal recording mode while the external field is applied along the *z*-axis). Furthermore, the experimental data on $m_{d}$ are commonly normalized to the mass of the specimen so that the recorded dipole moment, $m_{d}$, is eventually reported in units of emu/g. Accordingly, to make our theoretical relations (67) and (68) compatible to experimental data, we have to adjust the volume of the specimen, $V^{SHS}$, to the one that refers to the mass of 1 g. Obviously, this should be done by introducing the mass density, $\rho^{SHS}$, of the specimen. By following these maneuvers and by careful adjustment of the units, relation (68) obtains the following form

(69) $$m_{d}^{SHS}=C^{\left(1\right)}\chi_{m}\frac{H_{0}}{4\pi \rho^{SHS}}\hat{z}$$

In this relation the dipole moment, $m_{d}^{SHS}$, the external magnetic field, $H_{0}$, and the mass density, $\rho^{SHS}$, refer exclusively to experimental data drawn from the literature, while $C^{\left(1\right)}\chi_{m}$ is a correction factor originating from our model. Accordingly, we rewrite the above relation in the following more convenient, scalar form:

(70) $$\frac{m_{d}^{SHS}4\pi \rho^{SHS}}{H_{0}}=C^{\left(1\right)}\chi_{m}$$

We stress that both relations (69) and (70) are dimensionless and ready to use. This means that the value of the external magnetic field, $H_{0}$ (originally reported in units of Øe), and of the density, $\rho^{SHS}$ (originally reported in units of 10^6^ g/m^3^), should be inserted in these relations without using the respective units.

Once we have isolated the correction factor, $C^{\left(1\right)}\chi_{m}$, to the right side of relation (70) we can simulate its behavior in great detail. We recall that the coefficient, $C^{\left(1\right)}$, is given by relation (46) for l=1. Here we reproduce the correction factor for the sake of convenience

(71) $$C^{\left(1\right)}\chi_{m}=\frac{\chi_{m}}{\left(1+N_{1}\chi_{m}\left(1-N_{1+1}\chi_{m,1+1}^{ext}{\left(\frac{a}{b}\right)}^{3}\right)\right)}$$

**Figure 7:**
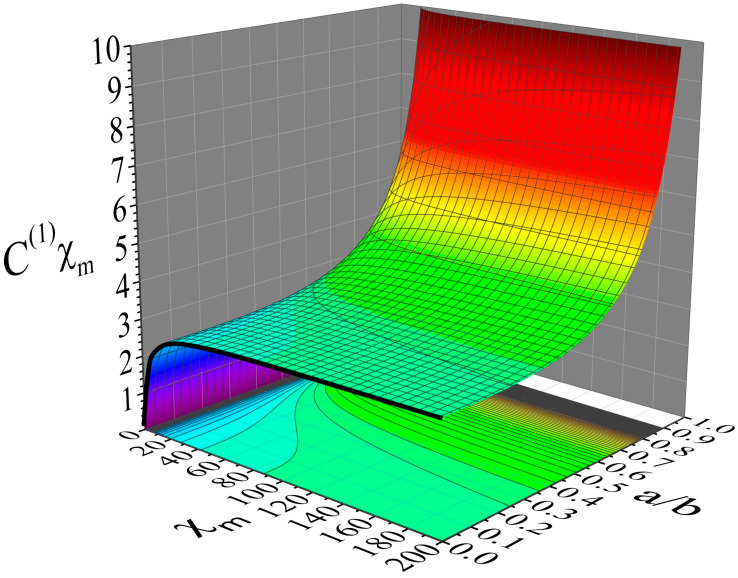
Simulations of the correction coefficient, $C^{\left(1\right)}\chi_{m}$, of relation (71) for a magnetic spherical hollow shell of susceptibility, $\chi_{m}$, inner radius a and outer radius b. The simulations cover the ranges $0\leq \chi_{m}\leq 200$ and 0≤a/b≤1 and refer to the basic mode, $U_{ext}^{\left(1,0\right)}(r)$, of the external potential, $U_{ext}(r)$. The inset thick-black curve, at the front of the simulated surface, describes the behavior of the solid sphere obtained when we set a=0 into relation (71) that describes the spherical hollow shell.

Figure 7 shows detailed simulations of the correction coefficient, $C^{\left(1\right)}\chi_{m}$, relation (71), in the entire range of radii ratio, 0≤a/b≤1, while the intrinsic susceptibility, $\chi_{m}$, has values within the range $0\leq \chi_{m}\leq 200$, covering a great variety of magnetic materials. The inset shows the conventional curve referring to the typical relation, $\chi_{m}^{ext}=\chi_{m}/(1+N\chi_{m})$, that describes a SS.

We see that the surface simulated in the above Figure 7 is practically flat over a relatively wide range of values of the susceptibility, $\chi_{m}$, and radii ratio, a/b, i.e., within $50\leq \chi_{m}\leq 200$ and 0≤a/b≤0.4. Accordingly, in this region where the experimentally determined factor, $m_{d}^{SHS}4\pi \rho^{SHS}/H_{0}$, is effectively constant (see relation (70)), it is difficult to discern any differences among distinct magnetic materials. This fact explains the difficulty to obtain the intrinsic susceptibility, $\chi_{m}$, from magnetization measurements when it attains relatively high values (see below). Obviously, this stems from the degeneration of the surface simulated by relation (71), referring to a spherical hollow shell, into the inset thick-black curve simulated by the relation, $\chi_{m}^{ext}=\chi_{m}/(1+N\chi_{m})$, that describes a solid sphere. The latter is obtained when we set a=0 into relation (71).

Below we make a comparison of the above simulations with relevant experimental data reported in the literature on specimens of magnetite, Fe_3_O_4_, spherical hollow shells. To this end, we need information on the intrinsic magnetic susceptibility, $\chi_{m}$, and mass density, $\rho^{SHS}$, of magnetite, for instance reported in [78,79,80,81,82], respectively.

In [79] magnetite, Fe_3_O_4_, was studied, among other forms, in single crystals having the ideal form of a sphere. The obtained extrinsic susceptibility, $\chi_{m}^{ext}=\chi_{m}/(1+N\chi_{m})$, was limited due to the demagnetization factor, N=1/3, to the maximum value, $\chi_{m}^{ext}=1/N=3$, that possibly can be recorded experimentally for the case of a sphere [44,45,64]. Accordingly, the value of the intrinsic susceptibility, $\chi_{m}=\chi_{m}^{ext}/(1-N\chi_{m}^{ext})$, could not be estimated reliably from the extrinsic one, $\chi_{m}^{ext}$. Obviously, when $\chi_{m}$ attains high values, the uncertainty originating from the relation, $1/\chi_{m}^{ext}=1/\chi_{m}+N$, is high so that the value of the intrinsic susceptibility, $\chi_{m}$, should be extracted with great caution from the experimentally obtained extrinsic one, $\chi_{m}^{ext}$. In [80] a great number of synthetic and natural magnetite crystals were investigated in sizes covering a great range, from 0.1 μm to 6 mm. In specimens approximated by a sphere the experimentally obtained extrinsic susceptibility, $\chi_{m}^{ext}$ (termed ‘initial’ in [80]), could be described by the demagnetization factor, N=1/3, and intrinsic susceptibility, $\chi_{m}$, given by the relation $\chi_{m}=\chi_{m}^{ext}/(1-N\chi_{m}^{ext})$. In such specimens the extracted $\chi_{m}$ attained values even exceeding 200. It should be noted that careful review of the literature evidences that there is a wide dispersion in the reported values of the intrinsic susceptibility, $\chi_{m}$, of magnetite, depending on the specific characteristics of each specimen.

In [78] hollow nanospheres of magnetite were prepared with diameter 460 nm and wall thickness 80 nm for utilization in microwave absorption applications. The calculated mass density of these hollow nanospheres was ρ = 3.78 g/cm^3^. In [81] a composite material of low density was developed in the form of glass-core/magnetite-shell spheres. This was achieved by coating glass microspheres with magnetite nanoparticles, of size smaller than 25 nm, via a process of chemical deposition. The authors succeeded to deposit a uniform and continuous coating of magnetite with thickness 400–500 nm over the surface of the glass microspheres. The mass density of this composite material was as low as ρ = 0.4–0.5 g/cm^3^. In [82] magnetite hollow particles of diameter 200–300 nm were prepared and ultimately were suspended in silicone oil for the construction of a magnetorheological colloidal fluid that was responsive to an external magnetic field. The mesoporous structure of the magnetite hollow particles was confirmed by means of gas porosimetry measurements that resulted in a mass density ρ = 4.37 g/cm^3^. The fact that magnetite in the form of spherical hollow shells has smaller mass density in comparison to that of bulk solid spheres, ρ = 5.2 g/cm^3^, is obvious. This is actually evident, at least indirectly, by almost all scanning electron microscopy and transmission electron microscopy data reported in the above publications of the literature. These data show that in almost all cases the magnetite spherical hollow shells have a granular/porous microstructure that should definitely relate to a lower mass density in respect to that of the bulk solid spheres.

Now we can perform a comparison of relations (70)–(71) and of the simulations shown in Figure 7 with detailed experimental results reported in the literature on the magnetization and structure of magnetite spherical hollow specimens [72,73,74,75,76,77,78]. To this end, for each case we should calculate the term of the left side of relation (70) and compare it either numerically to the correction factor of relation (71) or graphically to the simulated surface shown in Figure 7. This is done below for each case drawn from the literature. We note that, when needed, a digitizer was used for recovering reliable values from the magnetization curves [83]. Importantly, while surveying the magnetization data we restricted to the range of low values of the external magnetic field where an almost linear behavior is observed (or at least can be assumed to hold).

In [72,73] Sarkar et al. reported detailed data on magnetite nano-hollow spheres of various radii ratio, a/b. For instance, the specimen termed ‘Set 5’ in [72] (‘NHS-5’ in [73]) had a radii ratio a/b=0.58, and exhibited a dipole moment $m_{d}^{SHS}=32\;emu/g$, at an external field $H_{0}=500\;\emptyset e$ (sample ‘725 nm’ in Figure 7b of [72]). By using the bulk mass density, $\rho^{SHS}=5.2\;\times 10^{6}\;g/m^{3}$, the term of the left side of relation (70) obtains the value $m_{d}^{SHS}4\pi \rho^{SHS}/H_{0}=4.18$. When a lower density is used, e.g., $\rho^{SHS}=4.5\;\times 10^{6}\;g/m^{3}$, we obtain $m_{d}^{SHS}4\pi \rho^{SHS}/H_{0}=3.62$. The latter value fits nicely with the simulated surface of Figure 7 for an intrinsic susceptibility within the reasonable range, $103\leq \chi_{m}\leq 120$ (when the correction factor ranges approximately within, $3.61\leq C^{\left(1\right)}\chi_{m}\leq 3.63$).

In [74] Hu et al. fabricated monodisperse magnetite spherical hollow shells and reported detailed data on their radii ratio, a/b=0.70, and their dipole moment, $m_{d}^{SHS}=50\;emu/g$, attained at an external field, $H_{0}=732\;\emptyset e$ (Figure 9 in [74]). By using the bulk mass density, $\rho^{SHS}=5.2\;\times 10^{6}\;g/m^{3}$, the term of the left side of relation (70) obtains the value $m_{d}^{SHS}4\pi \rho^{SHS}/H_{0}=4.46$. When a lower density is used, e.g., $\rho^{SHS}=4.5\;\times 10^{6}\;g/m^{3}$, we obtain $m_{d}^{SHS}4\pi \rho^{SHS}/H_{0}=3.86$. These values fit fairly well with the simulated surface of Figure 7 even for the bulk mass density for an intrinsic susceptibility within the reasonable range, $128\leq \chi_{m}\leq 143$ (when the correction factor ranges approximately within, $4.45\leq C^{\left(1\right)}\chi_{m}\leq 4.47$).

In [75] Zhang et al. reported experimental data on the structure and electromagnetic properties of magnetite hollow nanospheres in single-crystalline form. The hollow nanospheres had a radii ratio a/b=0.73, and attained a dipole moment $m_{d}^{SHS}=54\;emu/g$, at an external field $H_{0}=900\;\emptyset e$ (Figure 5 in [75]). By using the bulk mass density, $\rho^{SHS}=5.2\;\times 10^{6}\;g/m^{3}$, the term of the left side of relation (70) obtains the value $m_{d}^{SHS}4\pi \rho^{SHS}/H_{0}=3.92$. When a lower density is used, e.g., $\rho^{SHS}=4.5\;\times 10^{6}\;g/m^{3}$, we obtain $m_{d}^{SHS}4\pi \rho^{SHS}/H_{0}=3.39$. Both values fit nicely with the simulated surface of Figure 7. For instance, even for the bulk mass density the intrinsic susceptibility attains values within the reasonable and relatively narrow range, $23\leq \chi_{m}\leq 24$ (when the correction factor ranges approximately within, $3.90\leq C^{\left(1\right)}\chi_{m}\leq 3.94$).

In [76,77] Chiba et al. and Kobayashi et al. reported detailed experimental data on magnetite submicron hollow spheres of various radii ratio, a/b. For instance, the sample ‘H420’ had a/b=0.50, and attained a dipole moment $m_{d}^{SHS}=24\;emu/g$, at an external field $H_{0}=350\;\emptyset e$ (Figure 1b in [77]). By using the bulk mass density, $\rho^{SHS}=5.2\;\times 10^{6}\;g/m^{3}$, the term of the left side of relation (70) obtains the value $m_{d}^{SHS}4\pi \rho^{SHS}/H_{0}=4.48$. When a lower density is used, e.g., $\rho^{SHS}=3.9\;\times 10^{6}\;g/m^{3}$, we obtain $m_{d}^{SHS}4\pi \rho^{SHS}/H_{0}=3.36$. The latter value fits nicely with the simulated surface of Figure 7 when the intrinsic susceptibility attains values within the reasonable and relatively wide range, $131\leq \chi_{m}\leq 168$ (for a correction factor ranging approximately within, $3.35\leq C^{\left(1\right)}\chi_{m}\leq 3.37$). Also, the sample ‘H720’ had a radii ratio a/b=0.31, and attained a dipole moment $m_{d}^{SHS}=21\;emu/g$, at an external field $H_{0}=350\;\emptyset e$ (Figure 1a in [77]). By using the bulk mass density, $\rho^{SHS}=5.2\;\times 10^{6}\;g/m^{3}$, the term of the left side of relation (70) obtains the value $m_{d}^{SHS}4\pi \rho^{SHS}/H_{0}=3.92$. When a lower density is used, e.g., $\rho^{SHS}=4\;\times 10^{6}\;g/m^{3}$, we obtain $m_{d}^{SHS}4\pi \rho^{SHS}/H_{0}=3.01$. The latter value fits nicely with the simulated surface of Figure 7 when the intrinsic susceptibility attains values within the reasonable and relatively wide range, $109\leq \chi_{m}\leq 144$ (for a correction factor ranging approximately within, $3.00\leq C^{\left(1\right)}\chi_{m}\leq 3.02$). We see that the spherical hollow shells of [77] exhibit a self-consisted behavior in respect to their mass density since they need a lower value, in comparison to other works of the literature, for obtaining quantitative agreement with our model.

In [78] Li and Yang reported experimental data on the absorption properties at microwaves for composites based on magnetite hollow nanospheres of radii ratio a/b=0.65 that attained a dipole moment $m_{d}^{SHS}=28\;emu/g$, at an external field $H_{0}=200\;\emptyset e$ (inset of Figure 3 in [78]). By using the bulk mass density, $\rho^{SHS}=5.2\;\times 10^{6}\;g/m^{3}$, the term of the left side of relation (70) obtains the value $m_{d}^{SHS}4\pi \rho^{SHS}/H_{0}=9.14$. When a significantly lower density is used, e.g., $\rho^{SHS}=2.3\;\times 10^{6}\;g/m^{3}$, we obtain $m_{d}^{SHS}4\pi \rho^{SHS}/H_{0}=4.04$. The latter value is able to fit the simulated surface of Figure 7 for a reasonable range of the intrinsic susceptibility, $124\leq \chi_{m}\leq 144$ (for a correction factor ranging approximately within, $4.03\leq C^{\left(1\right)}\chi_{m}\leq 4.05$). We note that this is the only case found in the literature where a significantly low value of the mass density was needed to obtain a fair quantitative comparison of our theoretical model with experimental data.

The detailed information presented in the above discussion on the conditions of optimum comparison between the experimental data drawn from the literature and the graphical means of Figure 7 is summarized in Table 1. The units in the specific form that should be employed are shown where needed. For each case we obtained the optimum range of values of the intrinsic susceptibility, $\chi_{m}$, that the magnetic spherical hollow shells employed in each reference should exhibit. Also, for each case we show the respective range of values of the correction factor, $C^{\left(1\right)}\chi_{m}$.

From the above extensive comparison of our theoretical results with detailed experimental data reported in the literature we conclude that our approach performs fairly well, given the complexity and the multiparametric nature of the investigated problem. Thus, it can be useful in the characterization of materials.

#### 6.1.6. Application to Shielding and Distortion for a Dielectric Spherical Hollow Shell

Returning to the dielectric spherical hollow shell, here we assess our analytical relations of the inside, 0≤r≤a, and outside, b≤r, spaces with respect to scattering in a complementary fashion to the discussion made above on the middle space, a≤r≤b. In this way we will validate our analytical relations throughout the entire space by assessing their reliability even among different applications.

Accordingly, here we discuss the shielding that can be achieved at the inside space (0≤r≤a) and the distortion that inevitably exists at the outside space (b≤r), on the basis of the utilization of the dielectric spherical hollow shell in applications. To this end, we recall the simulations presented in Figure 3 and Figure 6 for the inside and outside space, respectively, of the spherical hollow shell. From these simulations we inferred that there is a conflict between the shielding efficiency of the inside space from the external potential and the distortion that the latter experiences at the outside space. Indeed, these simulations evidenced that the intense elimination of the external potential, $U_{ext}(r)$, from the inside space will also result in its strong distortion at the outside space. Thus, it seems that in the dielectric spherical hollow shell we cannot have intense shielding of the inside space and negligible distortion at the outside space at the same time. This fact is of interest and deserves more attention since it can be useful in applications and also unveils the physical limitations of the spherical hollow shell as a primitive shield and/or scatterer.

Referring to the inside space, we recall that the transmission/shielding coefficients, $C^{\left(l\right)}$, relation (37), actually relate the general terms of the internal and external potentials through the relation $U_{int}^{\left(l,m\right)}\left(r\right)=\left(C^{\left(l\right)}-1\right)A_{ext}^{\left(l,m\right)}r^{l}Y_{l}^{m}(\theta ,\varphi )$, else, $C^{\left(l\right)}-1=U_{int}^{\left(l,m\right)}\left(r\right)/U_{ext}^{\left(l,m\right)}\left(r\right)$ (see relations (4), (5) and (35)–(37)). While coefficients, $C^{\left(l\right)}$, refer to the transmission/shielding of the inside space, we can define the new coefficients, $C^{\left(l\right)}-1$, as the distortion coefficients of the inside space, since, based on the above relation they quantify the distortion of the external potential, $U_{ext}^{\left(l,m\right)}\left(r\right)$, by the internal one, $U_{int}^{\left(l,m\right)}\left(r\right)$. Thus, we term these distortion coefficients of the inside space as $C_{dis}^{\left(l\right)}=C^{\left(l\right)}-1$, so that $C_{dis}^{\left(l\right)}=U_{int}^{\left(l,m\right)}\left(r\right)/U_{ext}^{\left(l,m\right)}\left(r\right)$. The latter relation reveals the physical meaning of these newly defined coefficients. In technical terms $C_{dis}^{\left(l\right)}$ are defined through the respective ones, $C^{\left(l\right)}$, by recalling relation (37). Notice that since $0\leq C^{\left(l\right)}\leq 1$ (see Figure 3), the newly defined coefficients range within $-1\leq C_{dis}^{\left(l\right)}\leq 0$. After some algebra we obtain the following relation for the distortion coefficients of the inside space:

(72) $$C_{dis}^{\left(l\right)}=-\frac{N_{l}N_{l+1}\chi_{e}^{2}\left(1-{\left(\frac{a}{b}\right)}^{2l+1}\right)}{\left(1+N_{l+1}\chi_{e}\right)\left(1+N_{l}\chi_{e}\left(1-N_{l+1}\chi_{e,l+1}^{ext}{\left(\frac{a}{b}\right)}^{2l+1}\right)\right)}$$

On physical grounds the above definition is in complete analogy to the distortion coefficients of the outside space, $D^{\left(l\right)}$, relation (40), that relate the general terms of the internal and external potentials through the relation $U_{int}^{\left(l,m\right)}\left(r\right)=D^{\left(l\right)}b^{2l+1}r^{-\left(l+1\right)}A_{ext}^{\left(l,m\right)}Y_{l}^{m}(\theta ,\varphi )$, else, $D^{\left(l\right)}={\left(r/b\right)}^{2l+1}(U_{int}^{\left(l,m\right)}\left(r\right)/U_{ext}^{\left(l,m\right)}\left(r\right))$ (see relations (4), (5) and (38)–(40)). The latter relation reveals the physical meaning of these distortion coefficients along with the fact that they should range within $-1\leq D^{\left(l\right)}\leq 0$ (see Figure 6). In technical terms $D^{\left(l\right)}$ are still defined through relation (40), reproduced here for the sake of convenience:

(73) $$D^{\left(l\right)}=-\frac{N_{l}\chi_{e}\left(1-{\left(\frac{a}{b}\right)}^{2l+1}\right)}{1+N_{l}\chi_{e}\left(1-N_{l+1}\chi_{e,l+1}^{ext}{\left(\frac{a}{b}\right)}^{2l+1}\right)}$$

Since both coefficients, $C_{dis}^{\left(l\right)}$ and $D^{\left(l\right)}$, have the same physical content, i.e., they quantify the distortion of the external potential, $U_{ext}^{\left(l,m\right)}\left(r\right)$, at the inside and outside space, respectively, their ratio can serve as an efficient parameter for the quantification of their relative impact on $U_{ext}^{\left(l,m\right)}\left(r\right)$. Through the above relations (72) and (73) the ratio, $C_{dis}^{\left(l\right)}/D^{\left(l\right)}$, termed as coefficients of relative distortion, $E_{rd}^{\left(l\right)}$, is easily obtained by the following relation:

(74) $$E_{rd}^{\left(l\right)}=\frac{C_{dis}^{\left(l\right)}}{D^{\left(l\right)}}=\frac{N_{l+1}\chi_{e}}{1+N_{l+1}\chi_{e}}$$

This relation is astonishingly simple. It evidences that these newly defined coefficients, $E_{rd}^{\left(l\right)}$, depend only on the degree, l, of the mode, (l,m), of the external potential, $U_{ext}^{\left(l,m\right)}\left(r\right)$ (however, not on its order, m) and on the endogenous properties of the LHI parent dielectric material, i.e., the intrinsic susceptibility, $\chi_{e}$. Unfortunately, the above relation proves that the coefficients, $E_{rd}^{\left(l\right)}$, do not depend on neither of the radii, inner r=a and outer r=b, of the spherical hollow shell. This is a significant disadvantage of the spherical hollow shell since it signifies that we do not have any geometrical degree of freedom, e.g., the radii ratio a/b, to selectively tune the relative distortion of the external potential at the inside space, 0≤r≤a, in comparison to that of the outside space, b≤r.

**Figure 8:**
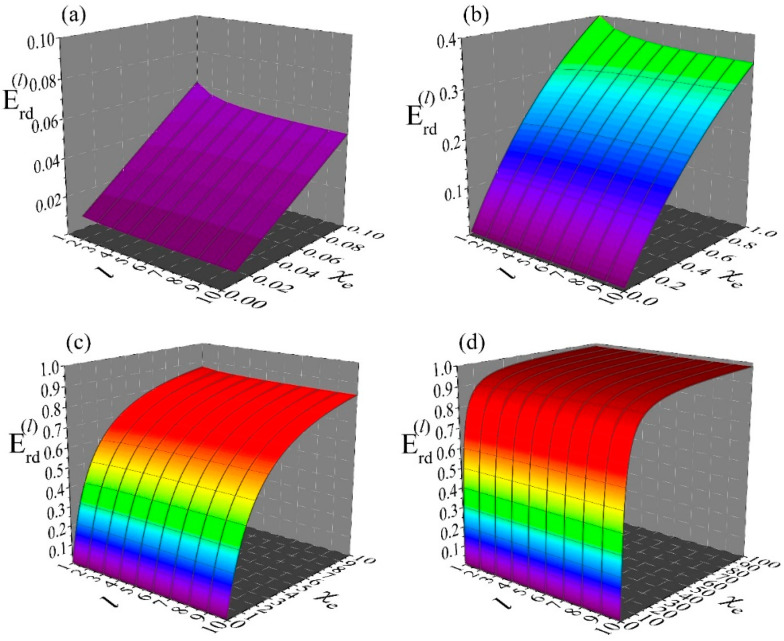
Simulations of the coefficients of relative distortion, $E_{rd}^{\left(l\right)}$, relation (74), for the first ten modes of the external potential, $U_{ext}^{\left(l,m\right)}\left(r\right)$, with degree 1≤l≤10 upon variation in the susceptibility, $\chi_{e}$, within an overall range $0\leq \chi_{e}\leq 100$. (**a**) Range of low values, $0\leq \chi_{e}\leq 0.1$. (**b**) Relatively increased range of values, $0\leq \chi_{e}\leq 1$. (**c**) Range of reasonable values, $0\leq \chi_{e}\leq 10$. (**d**) Overall range of high values, $0\leq \chi_{e}\leq 100$.

Figure 8 presents simulations of the coefficients of relative distortion, $E_{rd}^{\left(l\right)}$, based on relation (74). In all panels (a)–(d) the first ten modes of the external potential, $U_{ext}^{\left(l,m\right)}\left(r\right)$, with degree 1≤l≤10, are shown. The susceptibility, $\chi_{e}$, varies within an overall range, $0\leq \chi_{e}\leq 100$. Panel (a) focuses within a range of relatively low values, $0\leq \chi_{e}\leq 0.1$. The presented results show that in this range, of undesirably low values of $\chi_{e}$, the distortion observed at the inside space is indeed significantly lower than that of the outside space (however, this property is probably not useful for applications due to the extremely low values of $\chi_{e}$). In panel (b) we present data over an increased range of values, that however are still relatively low, $0\leq \chi_{e}\leq 1$. We see that now the distortion observed at the inside space becomes of the same order of magnitude to the distortion of the outside space. In panel (c) the values of $\chi_{e}$ cover a reasonable range, $0\leq \chi_{e}\leq 10$. We recall that the susceptibility, $\chi_{e}$, of most of the solids of everyday life falls within this range (see Table A1 of Appendix A in [38], and references therein). In this range of values the distortion observed at the inside space progressively becomes equal to that of the outside space. Finally, panel (d) covers the overall range extended to high values of $\chi_{e}$ within $0\leq \chi_{e}\leq 100$. Now the distortion observed at the inside space practically gets equal to that of the outside space. These detailed simulations prove that for the range of reasonable values of susceptibility, $\chi_{e}$, exhibited by most of the solids at ambient conditions, the distortion observed at the inside and outside spaces are of the same order of magnitude. Thus, for the case of the spherical hollow shell (constructed of linear, homogeneous and isotropic dielectric material) we cannot succeed intense shielding of the inside space, 0≤r≤a, from the external potential, $U_{ext}\left(r\right)$, without significant distortion of the latter at the outside space, b≤r (and vice versa). Simulations like the ones presented here can be used for referencing in applications, i.e., for the prescription of specifications on the response of the dielectric spherical hollow shell in respect to the desired inside-shielding versus the tolerable outside-distortion, in relation to the external potential, $U_{ext}\left(r\right)$.

## 7. Limitations and Perspectives

Here we investigated in detail the response of spherical hollow shells consisting of linear, homogeneous and isotropic dielectric and magnetic material, under the presence of an external potential/field of *any* spatial distribution. Importantly, our work refers to the static and quasistatic cases, that is to electric and magnetic potentials/fields of zero or of low frequency. This means that the introduction of properties that are important at increased frequencies (e.g., a dispersive or lossy behavior) to the constitutive relations of the studied materials and ultimately to the analytical relations obtained by our MLRS approach should be done very carefully, only at a very limited extent. Under such circumstances our analytical relations originating from the MLRS approach are useful, while any information on the dielectric and magnetic properties of the material can be introduced indirectly, e.g., through the incorporation of the frequency-dependent relative functions of dielectric permittivity, $\varepsilon_{r}(\omega )$, and magnetic permeability, $\mu_{r}(\omega )$, respectively. For instance, this was done in the theory of scattering where the static limit works nicely, having a significant predictive ability on the estimation of the resonance frequencies exhibited by spherical dielectric and magnetic scatterers. This was evidenced for the dielectric case in the above Section 6.1.4, “Application to Resonance Frequencies in Scattering by a Dielectric Spherical Hollow Shell”.

Also, the introduction of anisotropy can be done relatively easily, by still using the MLRS approach, given that the dielectric and magnetic material is still linear and homogeneous. Preliminary results show evidence that when the spherical hollow shell gets very thin, the polarization, whether electric or magnetic, exhibits a preferred angular orientation, i.e., over the surface, instead of radial one, i.e., through the surface, similar to the case of shape anisotropy. Significant problems in using the MLRS approach will arise when the material becomes non-linear and/or non-homogeneous. Then, the differential equations are difficult to solve, while analytical solutions can be drawn only for specific cases (e.g., when the inhomogeneity is only along the radial direction). Such cases can be investigated in future works.

Finally, the spherical hollow shell described in this work, even for the case of linear, homogeneous and isotropic materials, is a useful building unit for the construction of metamaterials and metasurfaces. In the case where these building units are placed far apart so that they do not interact, the mathematical description obtained here is still valid. However, in the case where the spherical hollow shells are brought close enough so that their interaction cannot be neglected, an entirely new strategy should be adopted from the beginning. In addition, when the spherical hollow shell consists of non-homogeneous material the typical Laplace equation is no longer valid within the volume of the shell. Instead, a Poisson-like equation will appear. In such cases the method of modeling by polynomials approximation [84,85] can possibly be used for obtaining the potential, the distribution of bound charges within the volume of the shell, etc.

## 8. Conclusions

Here we investigated in detail the response of spherical hollow shells, prone to depolarization processes due to their high surface area/volume ratio. These hollow shells consist of linear, homogeneous and isotropic dielectric and magnetic material and are subjected to an external potential, $U_{ext}\left(r\right)$, of *any* spatial form (either dc (static) or ac of low-frequency (quasistatic limit)). We applied the MLRS to the Laplace equation and calculated the internal and total potentials, $U_{int}\left(r\right)$ and $U_{tot}\left(r\right)$, self-consistently and generically, as an expansion of modes on the basis of SH. The solutions provided by the MLRS enabled us to directly obtain the expressions of the depolarization factor, for all different modes of the external potential, $U_{ext}\left(r\right)$, that possibly can be applied to spherical hollow shells in close connection to the transmission/shielding coefficients of the inside space and to the distortion coefficients of the outside space. Specifically, we identified two distinct depolarization factors, $N_{l}$ and $N_{l+1}$, accompanied by the relevant extrinsic susceptibilities, $\chi_{e,l}^{ext}$ and $\chi_{e,l+1}^{ext}$, that stem from the outer, r=b, and inner, r=a, surface of the spherical hollow shell, respectively. All $N_{l}$, $N_{l+1}$, $\chi_{e,l}^{ext}$ and $\chi_{e,l+1}^{ext}$ are degenerate on the order, m, of the mode of the external potential, $U_{ext}^{\left(l,m\right)}\left(r\right)$. Importantly, the sum of the two distinct depolarization factors equals unity, $N_{l}+N_{l+1}=1$, irrespective of the degree, l, of the mode of the external potential, $U_{ext}^{\left(l,m\right)}\left(r\right)$, as it should. The properties of the spherical hollow shells were investigated through detailed simulations. Also, detailed comparisons to real-life experiments were addressed to document the reliable applicability of our analytical results in materials science. The approach introduced here is generic and provides means to engineer the transmission to the inside space, the distortion to the outside space, and the polarization and depolarization effect of other dielectric and magnetic building units that are employed in applications.

## Figures and Tables

**Table 1:** Parameters involved in the comparison of our theoretical results with experimental data drawn from the literature (the upper index ‘SHS’ stands for ‘spherical hollow shell’).

Specimen/ Reference	$m_{d}^{SHS}/H_{0}$ (emu/g∅e)	$\rho^{SHS}$ $(10^{6}g/m^{3}$)	a/b	$m_{d}^{SHS}4\pi \rho^{SHS}/H_{0}$	$C^{\left(1\right)}\chi_{m}$	$\chi_{m}$
Set 5/[72,73]	32/500	4.5	0.58	3.62	3.61–3.63	103–120
n.a./[74]	50/732	5.2	0.70	4.46	4.45–4.47	128–143
n.a./[75]	54/900	5.2	0.73	3.92	3.90–3.94	23–24
H420/[76,77]	24/350	3.9	0.50	3.36	3.35–3.37	131–168
H720/[76,77]	21/350	4.0	0.31	3.01	3.00–3.02	109–144
n.a./[78]	28/200	2.3	0.65	4.04	4.03–4.05	124–144

## Data Availability

The original contributions presented in this study are included in the article. Further inquiries can be directed to the corresponding author.
